# A Rapid Scoping Review of Nutritional Management of Beef Cows and Pre-Weaning Calves and the Occurrence of Bovine Respiratory Disease in Pre- and Post-Weaning Calves in North America

**DOI:** 10.3390/ani16193116

**Published:** 2026-10-04

**Authors:** Barbara Wilhelm, Margarita Sanguinetti, Colleen Pollock, Cheryl L. Waldner

**Affiliations:** Department of Large Animal Clinical Sciences, Western College of Veterinary Medicine, University of Saskatchewan, Saskatoon, SK S7N 5B4, Canada

**Keywords:** beef cattle, bovine respiratory disease, cow-calf production, maternal nutrition, creep feeding, colostrum management, trace minerals, rapid scoping review

## Abstract

Bovine respiratory disease (BRD) causes economic loss and antimicrobial use in North American beef production. This rapid scoping review examined whether nutritional management of beef cows and pre-weaning calves affects BRD morbidity or mortality before or after weaning. Eligible sources were English- or French-language North American studies published from 2000 onward that evaluated nutrition or colostrum management in beef cows or calves and reported BRD-specific, secondary, or tertiary outcomes. Six bibliographic databases and grey-literature sources were searched. One reviewer charted and verified data using a pre-tested form. Findings were synthesized narratively without formal critical appraisal. Forty-seven studies were included: 22 reported BRD-specific morbidity or mortality, 20 reported only secondary outcomes (e.g., crude mortality), and five reported only tertiary outcomes (e.g., vaccine antibody titres). Three of five controlled trials of maternal protein supplementation reported favourable offspring BRD outcomes, although incomplete comparisons, absent treatment effect estimates, and the absence of formal critical appraisal limited interpretation. Selected maternal trace mineral trials reported reduced post-weaning BRD morbidity or fewer aggregated sick incidents. Direct evidence for colostrum-management strategies was sparse; other findings were inconsistent or based on few or no BRD events. Evidence remains insufficient for specific recommendations. Adequately powered commercial trials should report prespecified BRD outcomes, effect sizes, and confidence intervals.

## Simple Summary

Bovine respiratory disease is an important cause of illness, death, financial loss, and antibiotic use in North American beef production. Improving cow and calf nutrition could reduce disease risk, but the available evidence has not been systematically summarized. This rapid scoping review examined studies of maternal diets and supplements, calf creep feeding, vitamin and mineral injections, and colostrum management. Forty-seven studies were included: 22 reported BRD-specific morbidity or mortality, 20 reported only secondary outcomes (e.g., crude mortality), and five reported only tertiary outcomes (e.g., vaccine antibody titres). Several studies suggested that protein or trace mineral supplements provided to pregnant cows may improve respiratory health in their offspring. However, findings were inconsistent, respiratory disease occurred infrequently in many studies, and most trials provided insufficient statistical information to determine whether an intervention had no effect or the study was too small to detect one. Evidence supporting specific nutritional practices for preventing bovine respiratory disease is therefore limited and uncertain. Further commercial studies should include enough cattle and treatment-group replicates to detect meaningful differences and report the magnitude and precision of intervention effects. Better evidence could support recommendations that improve calf health, animal welfare, farm profitability, and responsible antibiotic use.

## 1. Introduction

Bovine respiratory disease (BRD) is a major economic cost to North American beef producers, due to production losses, treatment costs, and cattle mortality [1]. BRD is also a leading reason for antimicrobial use (AMU) in beef cow-calf operations and feedlots [2,3]. Because of the impact of BRD on the North American beef industry, risk factors for BRD and potential interventions to mitigate BRD risk have been investigated for decades [4]. The Five-Year Canadian Beef Research & Technology Transfer Strategy specifically endorses the development and promotion of cost-effective management practices and technologies that reduce the need for antibiotics to treat bovine disease such as BRD, including the assessment of how nutritional and health management of the cow herd impacts calf health pre- and post-weaning [5].

Post-natal calf nutrition in beef calves begins with colostrum. The importance of adequate colostrum consumption for reducing neonatal mortality risk in beef calves is well established, and the increased risk of neonatal mortality associated with failure of passive transfer (FPT) has been recognized for decades [6,7]. The potential effects of colostrum management procedures on the incidence of clinical BRD and on broader outcome measures such as pre- weaning morbidity and mortality have been investigated in a controlled trial [8]. A cross-sectional study has assessed nutritional and colostrum management strategies using surrogate or intermediate measures of passive transfer, such as neonatal serum immunoglobulin G (IgG) concentrations [9]. Researchers have also investigated associations between neonatal calf serum IgG concentrations in beef calves and health outcomes later in life, including pre- and post-weaning BRD [10,11]. Reviewing the potential effects of colostrum management on pre- or post-weaning BRD morbidity and mortality therefore requires the identification and synthesis of evidence from each of these study types.

Studies have examined whether supplementing beef cows or pre-weaning calves with trace minerals, including copper and selenium, reduces the risk of BRD, but findings have been inconsistent [12]. In an early controlled trial (CT), Muehlenbein et al. (2001) fed cows organic or inorganic copper from 45 days before calving to 60 days after calving and reported no effects on offspring health or performance [13]. In contrast, Grotelueschen et al. (2001) reported that supplementing beef cows with zinc, copper, manganese and cobalt during the summer grazing period significantly reduced the number of “sick incidents” among their offspring in the feedlot [14]. However, the supplement was not significantly associated with overall post-weaning BRD morbidity [14].

Several herd-level factors could influence the effectiveness of nutritional interventions in reducing BRD in beef calves, including the animals’ nutritional status before supplementation and the baseline frequency of BRD in the herd. Some studies have used supplement-delivery systems, such as individual feeding gates or hand-fed supplements delivered to grazing cows [15,16]; however, these systems are uncommon in commercial operations and may limit the applicability of the findings. Colostrum management practices could also modify the effects of interventions applied later in a calf’s life. More extensive winter-feeding systems, including swath, bale, and corn grazing, have become increasingly common in western Canadian beef production and can make micronutrient delivery and management more challenging [17]. These considerations raise questions about whether the published evidence supports practical recommendations for reducing BRD risk and whether the research settings reflect current beef-industry feeding practices.

Creep feeding, the practice of providing supplemental feed to calves to increase average daily gain and weaning weight, is often used to offset the effects of reduced pasture quality during drought or later in the grazing season. Because creep feeding may also influence calf nutrition and resilience around weaning, studies have examined its association with subsequent health outcomes. However, observational studies have reported inconsistent associations between creep feeding and subsequent BRD-specific or non-specific morbidity and mortality in beef calves [18,19,20]. Overall, across potential nutritional interventions, a knowledge gap exists regarding whether sufficient direct clinical evidence exists to recommend nutritional interventions to prevent BRD in calves.

A summary of the existing body of research on nutritional interventions affecting BRD morbidity or mortality requires the synthesis of studies with diverse designs that address multiple research questions across different settings. The Canadian Institutes of Health Research (CIHR) defines synthesis as “the contextualization and integration of research findings of individual research studies within the larger body of knowledge on the topic. A synthesis must be reproducible and transparent in its methods, using quantitative and/or qualitative methods” [21]. Scoping reviews are used when the purpose of the synthesis is to identify knowledge gaps, scope a body of literature, clarify concepts, or investigate how research has been conducted [22].

During the COVID-19 pandemic, the Cochrane Collaboration, a global leader in methodological guidance for research synthesis [23], drafted initial guidance for conducting rapid reviews, which was recently updated [24]. These guidelines provide a framework for conducting a rapid review in which some aspects of systematic review methodology are streamlined or omitted as appropriate for the specific project. More recently, the Cochrane Rapid Review Methods Group published reporting guidelines for rapid reviews that include three methodological items: reporting any iterative methods defined a priori and used during the review, the features that distinguish the rapid review from a traditional systematic review, and the involvement of knowledge users, such as policymakers, in the review process [25]. Rapid scoping reviews have been used to investigate human health questions, such as the relationship between antimicrobial use and COVID-19 [26] and the management of incidental findings in urgent care [27], whereas rapid scoping reviews of animal health questions have been reported less frequently [28].

The causal pathways between nutritional interventions and BRD morbidity and mortality may be complex, given the multiple components of some interventions, the multiple populations involved in intervention application and outcome assessment, and the potential for interactions among intervention components and between interventions and components of the production system. Guidance for assessing intervention complexity in reviews is available [29]. More recently, the Cochrane Collaboration has devoted an entire chapter of the Cochrane Handbook of Systematic Reviews of Interventions to intervention complexity [30]. The Handbook suggests three domains of complexity: the number of components in the intervention; interactions among intervention components or between the intervention and its context; and interactions with the wider system in which the intervention is introduced [30]. Some of the nutritional interventions described above include all three domains of complexity within a single study.

The broad question of whether nutritional management could mitigate BRD risk in North American beef calves is pertinent for the industry. Within this question, the objective of this rapid scoping review was to identify and describe North American studies of nutritional interventions in beef cows and pre-weaning calves and the occurrence of BRD in pre-weaning and post-weaning calves, including characterization of interventions investigated and settings in which these are applied. Two sub-questions further refined the review question. The first was to summarize the evidence on managing energy, protein, trace minerals, and other nutrients in beef cows or pre-weaning calves, including through creep feeding, and calf BRD morbidity and mortality before and after weaning. The second was to summarize the evidence on colostrum management in beef cows and BRD morbidity and mortality in their calves before and after weaning in North America. Another review objective was to identify knowledge gaps and the potential for future primary research or research synthesis. A protocol was developed using rapid-review methodological guidance, with Preferred Reporting Items for Systematic Reviews and Meta-Analyses (PRISMA) extension for scoping reviews PRISMA-ScR [31] as the primary reporting framework and PRISMA extension for complex interventions (PRISMA-CI) [32] as complementary guidance for intervention complexity and was deposited in the Digital Repository of the University of Saskatchewan (https://harvest.usask.ca/items/a002ce6a-b003-44de-93ff-bc75a0d532d1 accessed on 30 September 2026).

## 2. Materials and Methods

### 2.1. Review Approach and Management

Given the project timeline, a rapid scoping review approach was selected. Recent methodological recommendations for rapid reviews [24] were incorporated into the review methods.

- The funder convened an expert panel of key stakeholders to help define and refine the review questions.
- Date restrictions for inclusion (publications published from 2000 onward) were proposed to reflect major changes in beef production over the past several decades.
- Relevant systematic reviews were assessed, to allow capture of meta-analysis summary estimates of effect, where available. Systematic review reference lists were hand-searched as part of our scoping review search verification strategy.
- The electronic search strategy was developed from known relevant articles and previous reviews, then reviewed by subject-matter experts. Database-specific search strings, fields searched, search dates, and retrieval counts are reported in Supplementary File S1a.
- The protocol and report followed PRISMA-ScR, with PRISMA-CI used as complementary guidance for describing complex interventions [31,32]. Methodological streamlining incorporated in this review given the timeline included:
- Publication languages for included studies were limited to English or French, given project time constraints.
- For initial relevance screening, two reviewers (BW and MS) independently assessed a calibration sample of 50 citations and resolved disagreements. After calibration, one reviewer (BW) screened the remaining titles and abstracts using the questions, definitions, and promotion logic reported in Supplementary File S1b.
- Critical appraisal was not performed.
- Evidence was synthesized narratively.

### 2.2. Review Question, Scope, and Inclusion Criteria

#### 2.2.1. Broad Research Question

What are the characteristics of North American studies of nutritional interventions in beef cows and pre-weaning calves and the occurrence of BRD in pre-weaning and post-weaning calves, including characterization of interventions investigated and settings in which these are applied? The review question and sub-questions as presented in the protocol were slightly revised, congruent with the limitations of scoping review methodology in ranking interventions.

#### 2.2.2. Eligibility Criteria for Experimental Studies

**Population**—*Population receiving the intervention*: Included populations were beef cows at any production stage, including dry cows and cows with calves at foot, and beef calves from birth to weaning. Calves were considered weaned once they had been permanently separated from their dams. Studies conducted exclusively in dairy cattle, beef-on-dairy cattle, or calf-ranch settings were excluded.

*Population in which outcomes were assessed*: Studies measuring outcomes in beef calves from birth through slaughter were eligible. Studies measuring outcomes exclusively in beef-on-dairy cattle or cattle raised in calf-ranch settings were excluded.

**Intervention**—Any management or modification of nutrition through dietary supplementation, oral drenching, or injection was eligible. Relevant interventions included those involving energy, protein, water, vitamins, minerals, or colostrum.

**Comparators**—Eligible comparators included no intervention (negative control), an established standard of care, current practice, or an alternative intervention relevant to the nutritional strategy under investigation.

**Outcomes**—*Primary outcomes*: BRD-associated morbidity and BRD-associated mortality were the outcomes of greatest relevance to this review. Studies reporting one or both outcomes are hereafter referred to as studies reporting BRD-specific clinical outcomes. Outcomes described only as “treatment” were not classified as BRD-specific outcomes unless the authors explicitly indicated that treatment was administered for BRD.

*Secondary outcomes*: Secondary outcomes included crude or nonspecific morbidity, with the proportion attributable to BRD recorded where available; crude mortality, with the proportion attributable to BRD recorded where available; first-pull rates; and surrogate outcomes. Repull and retreatment rates were excluded because stakeholders indicated that these outcomes could vary substantially among settings because of differences in treatment protocols and staff training. Data from eligible studies reporting secondary outcomes were extracted and tabulated in the Supplementary Materials. Studies reporting secondary outcomes but no BRD-specific outcomes are hereafter referred to as studies reporting secondary outcomes only.

*Surrogate outcomes* were defined as physical or biological measurements occurring temporally between application of the nutritional intervention and assessment of a clinical BRD outcome. Examples included neonatal serum immunoglobulin, vitamin and trace mineral concentrations. These outcomes do not provide direct evidence of an association between nutritional intervention and BRD but may contribute to understanding the causal pathway linking the intervention to BRD.

*Tertiary outcomes*: Tertiary outcomes included immunological markers, such as humoral or cell-mediated responses to BRD vaccination and measures of innate immune responses relevant to BRD. Reports containing tertiary outcomes only were retained for evidence mapping and charted separately but were not included in the synthesis of BRD-specific or secondary clinical outcomes.

**Timing**—The timing and duration of each outcome-assessment period were recorded. Where possible, outcomes measured over the entire pre-weaning or post-weaning period were preferentially extracted and reported rather than outcomes measured during selected portions of these periods, consistent with stakeholder priorities.

*Exclusion based on outcome timing*: Studies in which outcomes were assessed exclusively during the neonatal period were excluded. The neonatal period was defined as birth through seven days of age, inclusive. The causes and antecedents of morbidity and mortality during this period differ from those occurring during the remainder of the pre-weaning period [33].

**Study design**—Eligible experimental study designs included randomized and nonrandomized controlled trials. Pertinent systematic and scoping reviews were flagged when identified by the search. Systematic review summary estimates of effect for relevant interventions on primary outcomes, were eligible for data extraction. Systematic and scoping reviews were used for search verification and to provide context for the captured primary research.

Data from eligible studies reporting BRD-specific outcomes were extracted and presented in the review tables and narrative synthesis.

#### 2.2.3. Eligibility Criteria for Observational Studies—Condition, Context and Population (CoCoPop)

**Condition**—Bovine respiratory disease was the condition of interest. It could be reported as the number of BRD cases or as BRD-specific morbidity or mortality risks or rates. BRD cases could also contribute to less specific outcomes, including feedlot pulls and crude morbidity or mortality risks or rates.

Data covering the entire pre-weaning or post-weaning period were preferred. Observational studies explicitly reporting BRD, pneumonia, respiratory disease, respiratory mortality, or respiratory treatment based on a defined case criterion were classified as studies reporting BRD-specific outcomes. Nonspecific morbidity, general antimicrobial treatment, undifferentiated first pull, and crude mortality were classified as secondary outcomes. First pull, repull, retreatment, general antimicrobial treatment, and BRD-specific treatment were charted as distinct outcomes; where reported separately, first pull was not combined with repull or retreatment.

Second pulls and retreatments for BRD were excluded as eligible secondary outcomes because stakeholders indicated that these measures could be highly setting-specific and could vary among feedlots because of differences in treatment protocols and staff training. When reported, first pull, repull, and retreatment remained distinct and were not combined. Studies examining nutritional management exclusively in relation to other health outcomes, such as neonatal calf diarrhea, were also excluded.

**Context**—*Contextual factors of primary interest*: Herd-level nutritional management practices and their reported associations with BRD in beef calves were contextual factors of primary interest. Observational studies that surveyed veterinarians rather than beef producers about producer practices were excluded, as were expert-elicitation studies. Data from eligible observational studies reporting contextual exposures of interest, such as creep feeding of calves, were extracted and presented in the review tables and narrative synthesis.

*Contextual factors of secondary interest*: Physical or biological surrogate measures for exposures identified in previous reviews and captured by the current search strategy were considered of secondary relevance when they had been associated with subsequent BRD in beef calves. Examples included neonatal serum immunoglobulin concentrations and observed or reported failure of passive transfer.

Studies of these exposures do not provide direct evidence of an association between nutritional interventions and BRD. However, they may improve understanding of potential causal pathways for BRD and identify priorities for future research. Data from eligible studies reporting secondary contextual factors were extracted and tabulated in the Supplementary Materials.

**Population**—The population eligibility criteria for observational studies were the same as those specified for experimental studies.

#### 2.2.4. Summary of Eligibility Criteria

Broadly, eligible primary sources included primary research published from 2000 onward in English or French that examined effects of nutritional management of cows or pre-weaned calves on BRD in pre- or post-weaning beef calves in North America. Exclusion criteria included studies conducted exclusively in dairy cattle, beef-on-dairy populations, or calf-ranch settings; studies published before 2000; studies conducted outside North America; reports not published in English or French; studies assessing outcomes exclusively during the neonatal period, defined as birth through seven days of age, inclusive; expert-elicitation studies; and surveys of human participants other than beef producers. Reports containing tertiary outcomes only were retained for evidence mapping and charted separately but were not included in the synthesis of BRD-specific or secondary clinical outcomes. Systematic and scoping reviews meeting the same topic criteria were retained separately as contextual sources rather than as primary studies; they were used for reference-list verification and, where available, for meta-analytic summary estimates cited in the Discussion.

### 2.3. Search Strategy

Search strategies were developed for AGRICOLA, MEDLINE, ProQuest Dissertations and Theses, PubMed, Scopus, and Web of Science. Search terms were identified from known relevant articles located during the initial scoping search and from search strategies reported in previous systematic reviews.

Terms were combined using the Boolean operator “OR” within categories representing the population, such as “calf”; intervention, such as “nutritional management”; and outcome, such as “pneumonia”. The categories were then combined using the Boolean operator “AND”. Complete database-specific search strategies are presented in Supplementary File S1a. The electronic searches were first conducted on 7 October 2025 and were updated on 30 August 2026 using the same databases, search concepts, and eligibility criteria.

The following sources were also hand-searched: The Bovine Practitioner, the annual conference proceedings of the American Association of Bovine Practitioners, and newsletters from meetings of the Academy of Veterinary Consultants, covering publications from 2000 through 2026. The database searches were supplemented by a Google search using the electronic search terms and by forward and backward citation searching of relevant key articles.

### 2.4. Relevance Screening

The level 1 relevance-screening form is presented in Supplementary File S1b. The form specifies questions addressing study design and setting, nutritional interventions, and eligible health outcomes, together with response options, operational definitions, and promotion logic. The two reviewers agreed on all 50 calibration decisions (100% observed agreement). Subsequently, one reviewer assessed the remaining 5435 records at this level. A random sample of 200 citations initially categorized as irrelevant was subsequently verified. No disagreements or potentially eligible studies were identified.

Following the calibration exercise, BW applied the form to the remaining titles and abstracts. Citations that met the promotion criteria or were classified as uncertain advanced to full-text relevance assessment.

### 2.5. Relevance Confirmation

The level 2 relevance-confirmation form is presented in Supplementary File S1c. One reviewer (BW) assessed all full-text reports against the review question and categorized them according to study design, intervention type, production stage at intervention application and outcome assessment, and reported outcome type. Ambiguous eligibility decisions were reviewed with one of the other authors of this review and resolved through discussion. Reports meeting the eligibility criteria and specified promotion logic advanced to data extraction.

### 2.6. Data Extraction

A pre-tested data-extraction form (Supplementary File S1d) was used to record study characteristics, intervention details, intervention-complexity variables, and outcome measures from each eligible report.

One reviewer (BW) extracted all data and subsequently verified the completed dataset against the source reports. Additionally, another reviewer (CW) independently verified a subset of data extraction from each included paper. No study investigators were contacted. Clinical outcomes of greatest relevance, specifically BRD morbidity and mortality, were preferentially extracted [24]. Data extraction also captured variables relevant to intervention complexity, including the components and timing of the intervention, the settings in which the intervention was applied, and outcome assessment, and potential effect modifiers, such as cattle genetics, vaccination status, and baseline nutritional status, when reported [29].

### 2.7. Data Analysis and Presentation

Data were compiled and narratively synthesized. Studies were grouped according to intervention type, production stage at intervention application, outcome type, and timing of outcome assessment. No formal critical appraisal or study-level risk-of-bias assessment was undertaken. Accordingly, the findings were not used to rank interventions according to comparative efficacy or certainty of evidence. Outcomes were interpreted within an explicit hierarchy: BRD-specific clinical outcomes, followed by nonspecific morbidity and mortality, then surrogate outcomes, and finally biological or immunological intermediates. Secondary and tertiary outcomes were used only to assess biological plausibility and were not used to strengthen conclusions about the prevention of clinical BRD. The review was managed using Covidence, a web-based systematic-review platform supported by the University of Saskatchewan (Covidence, Melbourne, Victoria, Australia).

The review was reported in accordance with PRISMA-ScR (checklist provided as Supplementary File S1e), with PRISMA-CI used as complementary guidance for reporting intervention and contextual complexity [31,32].

## 3. Results

### 3.1. Study Selection

**Figure 1:**
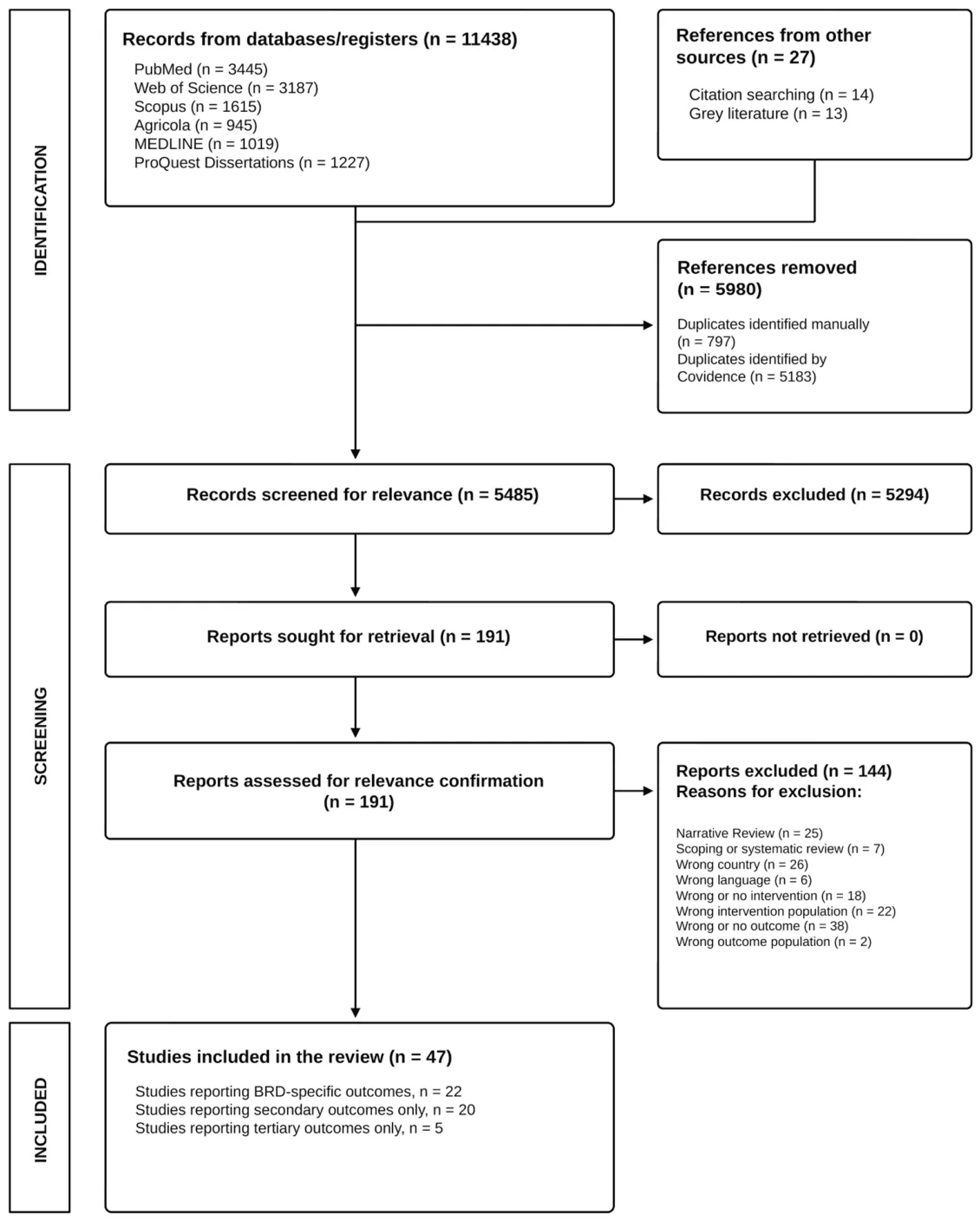
PRISMA flow diagram showing record identification, duplicate removal, title-and-abstract screening, full-text relevance confirmation, exclusions with reasons, and inclusion of studies in this rapid scoping review of nutritional management of beef cows and pre-weaning calves in relation to calf BRD morbidity and mortality before and after weaning in North America.

An initial scoping search conducted in PubMed in September 2025 identified several relevant systematic reviews [7,34,35], as well as controlled trials (CTs) investigating supplementation of cows [36], creep feeding of calves [37] and colostrum management [8]. Examination of the reference lists of these studies identified additional relevant studies and informed the development of three search-term groupings: nutritional management of cows, creep feeding of calves, and colostrum management. Searches were subsequently developed for selected electronic bibliographic databases using the following combination of term categories: Beef and Beef and (Population Terms) and (Intervention Terms) and (Outcome Terms).The electronic database searches were conducted on 7 October 2025 in AGRICOLA, MEDLINE, PubMed, Scopus, Web of Science, and ProQuest Dissertations and Theses. The documented database-specific search strategies, platform and field information, search dates, and retrieval results are presented in Supplementary File S1a. The initial searches identified 9438 records from six bibliographic databases and 27 records through other methods, including 14 through citation searching and 13 through grey-literature searching. An updated search using the same databases, search concepts, and eligibility criteria was conducted on 30 August 2026 and identified exactly 2000 additional database records. Across both searches, 11,438 database records and 27 records from other sources were identified. After removal of 5980 duplicate records, including 797 identified manually and 5183 identified by Covidence, 5485 records underwent title-and-abstract screening. Of these, 5294 were excluded, and 191 reports were sought for retrieval. All 191 reports were retrieved and assessed for relevance confirmation. Overall, 144 reports were excluded, leaving 47 studies included in this review (Figure 1). No multiple reports of the same underlying study were identified; therefore, the 47 included reports represented 47 unique studies. Of the included studies, 22 reported BRD-specific outcomes [8,14,15,16,18,19,20,36,37,38,39,40,41,42,43,44,45,46,47,48,49,50], which are summarized in Table 1. Of the 22 studies reporting BRD-associated outcomes, 7 also reported secondary outcomes (e.g., crude or nonspecific morbidity or mortality) [14,15,36,38,39,41,46].

A further twenty studies reported secondary outcomes only [9,10,11,13,51,52,53,54,55,56,57,58,59,60,61,62,63,64,65,66] and five reported tertiary outcomes only [67,68,69,70,71]; both groups are summarized in Supplementary Table S2. Reports containing tertiary outcomes only were retained for evidence mapping and charted separately but were not included in the synthesis of BRD-specific or secondary clinical outcomes.

Seven scoping or systematic reviews were also identified [7,34,35,72,73,74,75]. These reviews were not counted among the 47 included primary studies. Their reference lists were hand-searched to identify additional eligible primary studies, and their meta-analytic summary estimates were used as contextual evidence in the Discussion. The movement of records and reports through the review is shown in Figure 1.

### 3.2. Characteristics of Included Studies

Of the 47 included studies, 22 reported BRD-specific morbidity or mortality and are summarized in Table 1. The 20 studies reporting secondary outcomes only and the five reporting tertiary outcomes only are summarized in Supplementary Table S2.

Among the 22 studies reporting BRD-specific outcomes, one cross-sectional study [19] was conducted in western Canada, specifically Alberta, Saskatchewan, and Manitoba. The remaining 21 studies were conducted in the United States and were published between 2000 and 2025 (Table 1). The studies included 18 CTs, three cross-sectional studies, and one case–control study [8,14,15,16,18,19,20,36,37,38,39,40,41,42,43,44,45,46,47,48,49,50] (Table 1 and Supplementary Table S3). Nutritional interventions were applied to pre-weaning calves in six CTs [37,41,42,46,47,49] and examined as herd-level exposures in three cross-sectional studies [18,19,50]. These interventions included various creep-feed formulations. One additional CT examined the effects of colostrum management strategies applied to neonatal calves on pre-weaning BRD [8]. Nutritional interventions applied to cows were investigated in 11 CTs [14,15,16,36,38,39,40,43,44,45,48] and one case–control study [20]. Thirteen studies reported BRD morbidity or mortality before weaning, including 9 CTs [8,16,36,39,41,44,45,46,48] and four observational studies [18,19,20,50], whereas 15 CTs reported BRD outcomes after weaning [14,15,36,37,38,39,40,41,42,43,44,45,46,47,49] (Table 1).

All 18 CTs reporting BRD-specific outcomes applied the interventions to cows or pre-weaning calves on farms owned and managed by academic or research-oriented institutions, hereafter referred to as research cow-calf farms [8,14,15,16,36,37,38,39,40,41,42,43,44,45,46,47,48,49] (Table 2, Table 3 and Table 4). Fifteen of these CTs assessed BRD morbidity in post-weaning calves [14,15,36,37,38,39,40,41,42,43,44,45,46,47,49]: five conducted the post-weaning assessment in commercial feedlots [15,36,38,43,47], and ten conducted it in feedlots owned and managed by academic or research-oriented institutions, hereafter referred to as research feedlots [14,37,39,40,41,42,44,45,46,49]. Among the five CTs in which post-weaning outcomes were assessed in commercial feedlots, three reported statistically significant associations between the nutritional intervention and the outcome (*p* < 0.05) [15,36,38], whereas the other two reported no statistically significant associations [43,47]. Among the ten CTs that assessed post-weaning BRD morbidity in research feedlots, one reported a statistically significant association between the intervention and BRD, with greater morbidity in the control group [44].

Overall study-level incidence of post-weaning BRD in commercial feedlots ranged from 25.0% to 57.6%; the highest incidence reported for an individual treatment group was 67.7%. In research feedlots, the reported incidence ranged from 0% to 45%, although incidence was not consistently quantified: no cases were observed in studies [37,46]; an incidence of 45% was reported in study [14]; incidence was not reported in one study [44]; one case occurred among 42 calves in [41]; incidences of 12.1% and 9.1% were reported in studies [40,49], respectively; and morbidity was described as “minimal” in study [42].

### 3.3. Roughage, Macronutrient Supplementation, and Creep Feeding

Descriptive summaries of studies reporting BRD-specific outcomes for roughage, macronutrient, and creep-feeding interventions are presented in Table 2. Additional design characteristics of all included studies are presented in Supplementary Table S3, while studies reporting secondary outcomes only are summarized in Supplementary Table S4. Four CTs investigated maternal roughage or basal diets [39,44,62,63], five investigated protein supplementation of pregnant cows [15,38,40,44,45], and two investigated fat supplementation of pregnant cows [43,57]. Creep feeding of calves was examined in six CTs and three cross-sectional studies [18,19,37,41,42,46,47,49,50]. Of these studies, BRD-specific outcomes were reported by two of the four roughage or basal-diet CTs [39,44], all five protein-supplementation CTs [15,38,40,44,45], one of the two fat-supplementation CTs [43], and all nine creep-feeding studies [18,19,37,41,42,46,47,49,50].

**Table 2:** Descriptive summary of investigations of roughage, macronutrient, and creep-feeding studies reporting one or more BRD-specific outcomes.

Author, Year [Citation]	Intervention	Timing of Intervention Initiation (Duration)	Setting of Intervention Application (Setting of Outcome Assessment)	Population Outcome (Alternate BRD Morbidity Definition If Reported)	Results Comparator (Incidence of Outcome in Control or Comparator Group)	Treatment Effect (*p* Value)	Non-Clinical Health- Related Outcomes/ Comments
**ROUGHAGE SOURCES/BASAL DIETS FOR COWS**							
Larson et al., 2009 [44]	Winter range or corn residue	Late November to early March (Last trimester)	Res cow-calf ^1^ (Res feedlot ^1^)	Pre-weaning morbidity (BRD treatment)	Across treatment (tx) groups (gps) ^2^. (NR)	*p* = 0.68	NA
				Post-weaning morbidity (BRD treatment)	Across tx gps. (NR)	*p* = 0.83	
Radunz et al., 2012 [39]	Ad lib hay/ limit-fed corn/ dried corn distillers’ grains with solubles (DDGS)	Started on Day 167 gestation Year 1, D 155 Y2, stopped 1 week before calving	Res cow-calf (Res feedlot)	Post-weaning BRD morbidity at feedlot	Across tx gps. Energy source = hay, (8.90%)	*p* = 0.24	Neonatal calf serum IgG and failure of passive transfer stratified by dam diet Morbidity and mortality prior to feedlot arrival, i.e., birth to weaning plus 28 d backgrounding
			Res cow-calf (Res feedlot)	Post-weaning mortality at feedlot	Across tx gps. Hay ration, (0)	No mortality in any tx gp	
				Pre-weaning morbidity (Birth to weaning + 28 d backgrounding)	Across tx gps. Hay ration, (0)	*p* = 0.89	
				Pre-weaning mortality (Birth to weaning + 28 d backgrounding)	Across tx gps. Hay ration, (0)	*p* = 0.62	
**MACRONUTRIENT SUPPLEMENTS FOR COWS: CONTROLLED TRIALS**							
Larson et al., 2009 [44]	28% protein cubes with 62.0% DDGS, 10.6% wheat middlings, 9.0% cottonseed meal (CSM), 5.0% dried corn gluten feed, 5.0% molasses, 3.0% calcium carbonate, and 2.0% urea dry matter (DM), negative control ^3^	Late November to early March (Last trimester)	Res cow-calf (Res feedlot)	Pre-weaning BRD morbidity (BRD treatment)	Negative control, (NR)	*p* = 0.91	NA
				Post-weaning BRD morbidity (BRD treatment)	Negative control, (NR)	*p* = 0.05 Significantly increased control gp BRD	
Mulliniks et al., 2013 [38]	Pos control ^3^ = 36% crude protein (CP) (CSM + urea sup, or self-fed 28% CP corn gluten meal, DDG plus trace mineral sup, or negative control	60 d during late gestation, stopping 2 wk prior to calving (60 d)	Res cow-calf (Com feedlot ^1^)	Post- weaning BRD morbidity (Treated for respiratory disease)	Across tx gps. Negative control, (25%)	*p* = 0.05 Significant difference across tx gps with no cases in self-fed corn gluten meal gp	NA
				Post- weaning mortality	Across tx gps. Negative control (0)	*p* = 0.02 Significant difference across tx gps with 0 mortality neg control or SMP gp, 16% in CSM gp	
Mulliniks et al., 2012 [15]	Pos control ^3^ = 36% CP CSM protein-based cubed supplement hand-fed; self-fed blood and feather + trace mineral sup; neg control ^3^ CSM fed twice weekly when required, at 454 g/cow/d like pos control.	Intermittent CSM as deemed needed; hand-fed CSM and self-fed blood and feather meal 27, 62, 93 and 51 d Y1–4	Res cow-calf (Com feedlot)	Post-weaning BRD morbidity (Treated for respiratory disease)	Across tx gps. CSM as required, (37%)	*p* = 0.03 Significant difference across tx gps, with fewer cases in self-fed gp	NA
				Post-weaning mortality	Across tx gps, CSM as required (3.3%)	*p* = 0.89	
Shoup et al., 2015 [40]	Low (2.16 kg/cow/day) vs. high (8.61 kg/cow/day) dose of 70% DDGS; 30% soybean hull sup; negative control	103 d prepartum, stopped when calving half done (>103 d)	Res cow-calf (Res feedlot)	Post-weaning BRD morbidity (First BRD treatment)	Across tx gps. negative control, (12.1%)	*p* = 0.62	NA
				Post-weaning mortality	Across tx gps. negative control (6.1%)	*p* = 0.93	
Moriel et al., 2020 [45]	Sugarcane molasses and urea ± methionine hydroxy analogue, negative control	57 ± 5 d prepartum— 17 ± 5 d postpartum (72 d)	Res cow-calf (Res feedlot)	Pre-weaning BRD mortality	Across tx gps. Negative control; (0)	No mortality in any tx gp	Serum antibody titers against BVDV-1a and PI-3 viruses; plasma concentrations of glucose, insulin-like growth factors 1 and 2 (IGF-1 and IGF-2), l-methionine, methionine adenosyl transferase 1A (MAT1A), S-adenosyl methionine (SAM), glucose, cortisol, haptoglobin
			Res cow-calf (Res feedlot)	Pre- weaning BRD morbidity	Negative control; (0)	No morbidity in any tx gp	
Brandão et al., 2020 [43]	Calcium Salts of Soybean Oil (CSSO) = 415 g/cow/d soybean meal + 170 g/cow/d Ca salts of soybean oil (Essentiom™, Church and Dwight Co Inc, Princeton, NJ) Control = 415 g/cow/d soybean meal + 170 g/cow/d prilled fat (Energy Booster^TM^, Milk Specialties, Eden Prairie, MN) +25 g/d limestone	85 d prepartum (85 d)	Res cow-calf (Com feedlot)	Post- weaning BRD morbidity (Treatment once for respiratory disease)	Across tx gps. Prilled fat gp, (40.5%)	*p* = 0.16	Treatment twice for respiratory disease, number of treatments
**CASE-CONTROL STUDY**							
Woolums et al., 2018 ^4^ [20]	Supplement to cows	NR	Com cow-calf ^1^ (Com cow-calf)	Pre-weaning BRD morbidity	No supplement, (NA)	*p* > 0.20 in UV analysis	Univariable conditional logistic regression
**CREEP FEEDING CALVES**							
Arthington et al., 2008 [37]	Creep-feed supplement (15.1% CP, 78.3% TDN, 0.63% Ca; Ca:P ratio 1:1), negative control	45 d pre-weaning Y1, 53 d Y2 (Y1 = 45 d, Y2 = 53 d)	Res cow-calf (Res feedlot)	Post- weaning BRD morbidity	No creep feed (0)	No BRD morbidity in any tx gp	Acute phase proteins haptoglobin, ceruloplasmin
Palmer et al., 2021 [46]	Creep (17.30% CP) with yeast (4 g/d yeast (3 g of live yeast and 1 g of yeast cell wall product; Phileo by Lesaffre), fed at 0.5% of body weight, as-fed, OR Conventional 17.95% CP creep	35 d pre-weaning + 42 d backgrounding (77 d)	Res cow-calf (Res feedlot)	Pre- weaning BRD morbidity	Creep without yeast, (0)	No morbidity in any tx gp	WBC counts, *E. coli* phagocytosis, haptoglobin
			Res cow-calf (Res feedlot)	Post- weaning BRD morbidity	Creep without yeast, (0)	No morbidity in any tx gp	
			Res cow-calf (Res feedlot)	Pre- weaning BRD mortality	Creep without yeast, (0)	No mortality in any tx gp	
			Res cow-calf (Res feedlot)	Post-weaning mortality	Creep without yeast, (0)	No morbidity in any tx gp	
Schubach et al., 2019 [47]	Ca salts of soybean oil (CSSO; 80 g per steer of Essentium™) added to creep or 67 g per steer of prilled saturated fat (Energy Booster™)	60–120 d of age (60 d)	Res cow-calf (Com feedlot)	Post-weaning BRD morbidity during the 40 d post-weaning feeding period	Across tx arms. Creep with prilled fat (57.6%)	*p* = 0.56	NA
Shike et al., 2007 [41]	Fibre-based creep or conventional creep without fibre	63–189 d of age (126 d)	Res cow-calf (Res feedlot)	Pre-weaning BRD morbidity	Creep without fibre, (1/42)	“No differences in morbidity across tx gps”	NA
			Res cow-calf (Res feedlot)	Post-weaning mortality	Creep without fibre, (0) reported	No reported mortality in the conventional creep comparator; no reported between-group mortality	
Tjardes, 2020 [49]	Creep (30% corn gluten, 30% coarse cracked corn, 30% soybean hulls, 5% commercial vitamin and mineral balancer containing Rumensin, and 2.5% molasses), Negative control	21 d pre-wean (21 d)	Res cow-calf (Res feedlot)	Post- weaning BRD morbidity	No creep, (9.1%)	*p* = 0.98	Distinct morbidity outcome.
		21 d pre-wean (21 d)	Res cow-calf (Res feedlot)	Post-weaning BRD mortality	No creep, (0%)	Mortality was 0% in both tx gps	NA
Wright et al., 2000 [42]	Creep with trace minerals Expt 1 Control = Creep without trace minerals; Creep plus 500 IU Vit E + 0.3 mg/kg Se DM, Creep + 10 mg/kg Cu DM, Creep + 500 IU Vit E + 0.3 mg/kg Se + 10 mg/kg Cu DM. Expt 2 Control = Creep without trace minerals, Creep + 0.3 mg/kg Se DM; Creep + 500 IU Vit E + 0.3 mg/kg Se DM; Creep + 1000 IU Vit E + 0.3 mg/kg Se DM; Creep + 1500 IU Vit E + 0.3 mg/kg Se DM	Expt 1 49 d, Expt 2 53 d pre-wean (Expt 1 49 d, Expt 2 53 d)	Res cow-calf (Res feedlot)	Pre-weaning morbidity Post-weaning BRD morbidity (Anorexia, nasal discharge, excessive salivation, dyspnea, inflamed nares)	Creep without trace minerals (NR)	Across tx gps. *p* > 0.05	APP, ceruloplasmin, haptoglobin, lymphocyte blastogenic conc, BVD titres
**CREEP FEEDING: CROSS-SECTIONAL STUDIES**							
Hanzlicek et al., 2013 ^4^ [18]	Creep feed (Y/N)	Pre-weaning (NR)	Com cow-calf (Com cow-calf)	Pre-weaning BRD morbidity (Antimicrobial treatment for respiratory disease)	No creep feed, (NR)	Incidence rate ratio, *p* > 0.30; negative- binomial univariable analysis	NA
Waldner et al., 2022 ^4^ [19]	Creep feed (Y/N)	Pre-weaning (NR)	Com cow-calf (Com cow-calf)	Pre-weaning BRD morbidity	No creep, (NR)	*p* > 0.20 in UV analysis	NA; generalized estimating equations employing a binomial distribution and logit link function
Woolums et al., 2013 ^4^ [50]	Nursing calves fed supplemental feed, such as creep feed (yes/no)	Pre-weaning (NR)	Com cow-calf (Com cow-calf)	Cumulative incidence of treatment of nursing calves for BRD	No supplemental feed (Reference)	Adjusted relative risk 1.7 (95% CI 1.1 to 2.4); *p* = 0.007	Multivariable negative binomial component of a zero-inflated negative binomial model; unit of analysis: cow-calf operation (herd).
			Com cow-calf (Com cow-calf)	Pre-weaning BRD morbidity	No creep, (NR)	(Incidence rate ratio = 1.7 (1.4, 2.4) in operations experiencing BRD) *p* = 0.007	Multivariable zero-inflated negative binomial regression, accounting for number of cows in herd, region, calf introductions, estrous synchronization, or use of pregnancy diagnosis.

Some studies employed a factorial study design and therefore the same investigation may be cited presenting different contrasts across nutrient topic subheadings. Abbreviations: CP = crude protein; CSM = cottonseed meal; CSSO = calcium salts of soybean oil; d = day; DDG = dried distillers’ grains; DM = dry matter; Expt = experiment; Gp = group; mo = month; NA = not applicable; NR = not reported; rep = replicate; TDN = total digestible nutrients; tx = treatment; Y/N = yes/no; Y1 = Year 1. ^1^ Res cow-calf = research cow-calf farm, i.e., operated by university or research facility; Res feedlot = research feedlot; Com cow-calf = commercial cow-calf farm; Com feedlot = commercial feedlot. ^2^ Across tx gp = significance of difference in the outcome is assessed across multiple treatment or control groups as opposed to contrasts. ^3^ Pos control = positive control = treatment other than the one of primary interest; neg control = negative control = no intervention/treatment. ^4^ Observational studies. Hanzlicek et al. [18], Waldner et al., 2022 [19], and Woolums et al., 2013 [50] were cross-sectional studies. Woolums et al., 2018 [20] was a case–control study. Studies cited without superscript were controlled trials.

#### 3.3.1. Maternal Roughage and Basal Diets

One CT compared three gestational diets: hay offered free choice, limit-fed corn, and dried corn distillers’ grains with solubles (DDGS). The interventions began on day 167 of gestation in year 1 and day 155 in year 2 [39]. The incidence of respiratory disease among offspring in the feedlot did not differ significantly across treatment groups (*p* = 0.24). The study also found no statistically significant differences in crude pre-weaning morbidity or mortality among maternal treatment groups (*p* > 0.05) [39] (Table 2).

Similarly, Larson et al. (2009) compared cows maintained on winter range or corn residue during late gestation, from mid-November to mid-February, and found no statistically significant differences in pre- or post-weaning BRD morbidity among treatment groups [44] (Table 2). Two other CTs compared hay- and corn-based diets and reported no statistically significant differences in crude morbidity or mortality among offspring [62,63] (Supplementary Table S4). In the first of these studies, the corn-based diet provided 125% of the dams’ total digestible nutrient (TDN) requirements, whereas the hay-based ration provided 100% [62].

#### 3.3.2. Maternal Macronutrient Supplementation

Six CTs reporting BRD-specific outcomes investigated maternal macronutrient supplementation during gestation and subsequent BRD in offspring [15,38,40,43,44,45]. Three reported statistically significant differences in post-weaning BRD morbidity among treatment groups [15,38,44] (Table 2). In a U.S. study, Brandão et al. (2020) compared supplementation of beef cows with calcium salts of soybean oil or prilled fat for 85 days before calving and found no statistically significant difference in post-weaning BRD morbidity among offspring (*p* > 0.05) [43] (Table 2). In a Canadian CT reporting a secondary outcome, cows received calcium salts of palm oil, flaxseed, or no fat supplement from 1 month before calving until turnout. Crude pre-weaning mortality was significantly greater in the un-supplemented control group (*p* = 0.02) [57] (Supplementary Table S4).

Five CTs reporting BRD-specific outcomes investigated protein supplementation of pregnant beef cows [15,38,40,44,45] (Table 2). Larson et al. (2009) fed cows cubes containing 28% crude protein (CP) from late November to early March and reported significantly more post-weaning BRD cases among offspring in the un-supplemented control group (*p* = 0.05) [44]. Mulliniks et al. (2013) compared three treatments administered for 60 days, from December until 2 weeks before the start of calving: a hand-fed supplement containing 36% CP from cottonseed meal and urea; a self-fed supplement containing 28% CP from corn gluten meal and dried distillers’ grains (DDG), with added trace minerals; and no protein supplement [38]. No post-weaning BRD treatments were reported among offspring of cows receiving the self-fed supplement [38]. Mulliniks et al. (2012) also compared three supplementation strategies: a hand-fed, cottonseed meal-based cubed supplement containing 36% CP; a self-fed supplement containing blood meal, feather meal, and trace minerals; and cottonseed meal provided twice weekly, when conditions required, at a rate equivalent to 454 g/cow/day [15]. The authors reported fewer post-weaning treatments for respiratory disease among offspring of cows receiving the self-fed supplement (*p* = 0.03) [15]. Shoup et al. (2015) [40] compared low- and high-level supplementation of prepartum beef cows at 2.16 and 8.61 kg/cow/day, respectively. The supplement contained 70% dried distillers’ grains with solubles (DDGS) and 30% soybean hulls [40]. Moriel et al. (2020) compared a sugarcane molasses and urea supplement provided with or without methionine hydroxy analogue from 57 days before calving to 17 days after calving [45].

Of the five CTs investigating maternal protein supplementation, three reported statistically significant differences in post-weaning BRD morbidity among treatment groups [15,38,44]; in each study, one supplementation strategy was associated with lower BRD morbidity than the control or another treatment strategy. Moriel et al. (2020) reported no pre-weaning morbidity or mortality in any treatment group [45], whereas Shoup et al. (2015) found no statistically significant differences in post-weaning BRD morbidity or mortality among treatment groups [40].

In a study reporting secondary outcomes, Wilson et al. found no statistically significant difference in crude post-weaning morbidity among offspring of fall-calving cows that did or did not receive 2.1 kg/cow/day of DDGS during late gestation [64] (Supplementary Table S4). A U.S. case–control study also found no statistically significant association between feeding unspecified “cow supplements” and the herd-level outcome of treating more than 5% of nursing calves for BRD annually (*p* > 0.20). This study included beef herds in Nebraska, North Dakota, and South Dakota [20] (Table 2).

Post-weaning BRD morbidity was reported in six CTs of maternal macronutrient supplementation [15,38,40,43,44,45]. In three studies that assessed health outcomes in commercial feedlots [15,38,43], BRD incidence in the control groups ranged from 25.0% to 40.5%. In the other three studies, which assessed outcomes in research feedlots [40,44,45], BRD incidence in the control groups ranged from 0% to 12.1% (Table 2).

#### 3.3.3. Creep Feeding

Creep feeding of pre-weaning beef calves was examined in six CTs [37,41,42,46,47,49] and three cross-sectional studies [18,19,50], all of which reported BRD-specific outcomes. The CTs evaluated creep feeds containing various ingredients, including yeast and calcium salts of soybean oil (Table 2). None of the CTs detected statistically significant differences in BRD morbidity or mortality among treatment groups (*p* > 0.05). However, two CTs reported no morbidity or mortality in any treatment group during the study period [37,46].

The cross-sectional studies found no statistically significant association between producer-reported creep feeding and the odds that a herd reported pre-weaning BRD morbidity. However, among herds reporting BRD in nursing calves, Woolums et al. (2013) found that providing supplemental feed to calves, such as creep feed that was inaccessible to cows, was associated with a greater cumulative incidence of calves treated for BRD (Relative Risk = 1.7, 95% CI: 1.1–2.4; *p* = 0.007) [50].

BRD incidence was low in many studies of roughage, macronutrient supplementation, or creep feeding. Among creep-feeding studies conducted in research feedlots, two reported no post-weaning BRD cases [37,46], and one described morbidity as “minimal” [42]. Two other creep-feeding studies quantified post-weaning BRD incidence: one reported an incidence of 9.1% in a research feedlot [49], whereas the other reported an incidence of 57.6% in a commercial feedlot [47].

Across the CTs investigating roughage, macronutrients, or creep feeding, no estimates of treatment effect magnitude or precision were reported for BRD-specific or secondary health outcomes.

### 3.4. Trace Mineral Supplementation

Overall, trace mineral supplementation of cows was investigated in ten CTs [13,14,16,36,48,51,53,54,59,66], including four that reported BRD-specific outcomes [14,16,36,48] and six that reported secondary outcomes only [13,51,53,54,59,66]. Vitamin or trace mineral interventions in calves were investigated in four studies [19,42,61,65]: one CT of supplemented creep feed reporting BRD-specific outcomes [42], one CT of injectable trace minerals reporting secondary outcomes only [65], and two cross-sectional studies, one reporting BRD-specific outcomes and one reporting secondary outcomes only [19,61]. The four CTs [14,16,36,48] and one cross-sectional study [19] reporting BRD-specific outcomes are summarized in Table 3. Studies of trace mineral interventions reporting secondary outcomes only are summarized in Supplementary Table S5.

**Table 3:** Descriptive summary of investigations of trace minerals and vitamins reporting BRD-specific outcomes.

Author, Year [Citation]	Intervention	Timing of Intervention Initiation (Duration)	Setting of Intervention Application (Setting of Outcome Assessment)	Population Outcome (Alternate BRD Morbidity Definition If Reported)	Comparator (Incidence of Outcome in Control or Selected Comparator Group)	Treatment Effect (*p* Value)	Other Outcomes/ Comments
**SELENIUM SUPPLEMENTS TO COWS**							
Patterson et al., 2013 [16]	Organic, inorganic, or a 1:1 blend of organic and inorganic selenium in a free-choice mineral mix with 35 ppm selenium	Beginning at six to eight months of gestation (Approximately one year)	Research cow-calf pasture (Research cow-calf pasture, birth through weaning)	Pre-weaning calf BRD mortality	Comparison across the three selenium-source groups	No BRD deaths occurred in any treatment group; therefore, no comparative effect was estimable	One calf in the mixed-source selenium group died at birth; this death was distinct from the BRD-mortality outcome.
**COPPER AND OTHER TRACE MINERAL SUPPLEMENTS TO COWS**							
Grotelueschen et al., 2001 [14]	Control mineral = salt-dicalcium phosphate-based mixture; metal-complex mineral with added Cu, Mn, Zn, Co; Sulfate-based inorganic mineral with added sulfate-based Cu, Mn, Zn, Co.	4 June to 19–25 September (Approximately 110 d)	Res cow-calf (Res feedlot)	Post-weaning BRD morbidity	Across tx gps ^2^ Basal diet, no supplement (45.0%)	*p* > 0.05	“Sick incidents,” i.e., total of first-time tx, retreatments and re-pulls - response to *Mannheimia haemolytica* and viral vaccines
				Post-weaning mortality	Basal diet, no supplement (3.01%)	*p* > 0.05	
Marques et al., 2016 [36]	Control = no supplemental Cu, Co, Mn, and Zn; Sulfate sources of Cu, Co, Mn, and Zn, or organically complexed source of Cu, Co, Mn, and Zn.	93–99 d prepartum (93–99 d)	Res cow-calf (Com feedlot ^1^)	Post-weaning BRD morbidity Pre-conditioning phase	Across tx gps Basal diet, no supplement (34.9%)	No significant difference across tx gps *p* = 0.95	Post- weaning mortality
				Post-weaning BRD morbidity Grower phase	Across tx gps Basal diet, no supplement (42.3%)	*p* = 0.02 BRD morbidity was lower in organic tx gp	
				Post-weaning BRD morbidity, feedlot phase	Across tx gps Basal diet, no supplement (0)	No significant difference across tx gps; *p* = 0.37	
				Post-weaning mortality, preconditioning phase	Basal diet, no supplement (0)	*p* = 0.42	
				Post-weaning mortality, grower phase	Basal diet, no supplement (9.9%)	*p* = 0.63	Percentage of calves slaughtered per cow did not differ among groups; overall post-weaning mortality was similar
				Post-weaning mortality, feedlot phase	Basal diet, no supplement (0)	No mortality in any group	
Stephenson et al., 2023 [48]	Control = no supplemental Cu, Mn, or Zn; Sulfate-based Cu, Mn, Zn; Metal methionine hydroxy analogue-based Cu, Mn, Zn; or Sulfate-based and methionine hydroxy analogue combined.	91.2 ± 6.2 d pre-calving to 11 ± 3 d post-calving (102 d)	Res cow-calf (Res cow-calf)	Pre-weaning BRD morbidity	Across tx gps Basal diet, no supplement (0)	No significant difference across tx gps; minimal morbidity during study period (1 calf from 1 gp)	Serum proteins including IgG, colostrum composition
**TRACE MINERALS AND VITAMINS TO CALVES**							
Waldner et al., 2022 ^3,4^ [19]	Vitamin E/selenium injection near birth	Near birth	Com cow-calf ^1^	Pre-weaning BRD treatment	No vitamin E/ selenium injection near birth	Not retained in published final multivariable BRD models; no adjusted estimate reported.	
	Vitamin A/D injection near birth	Near birth	Com cow-calf	Pre-weaning BRD treatment	No vitamin A/D injection near birth	Not retained in published final multivariable BRD models; no adjusted estimate reported.	

Note: Wright et al., 2000 [42] investigated creep with various levels of trace minerals and is presented in Table 2. Abbreviations: AM = antimicrobial; d = day; gp = group; mo = month; NA = not applicable; rep = replicate; tx = treatment; Y/N = yes/no; Y1 = Year 1. ^1^ Res cow-calf = research cow-calf farm, i.e., operated by university or research facility; Res feedlot = research feedlot; Com cow-calf = commercial cow-calf farm; Com feedlot = commercial feedlot ^2^ Across tx gp = significance of difference in the outcome is assessed across multiple treatment or control groups as opposed to contrasts. ^3^ Cross-sectional study. Studies cited without superscript were controlled trials. ^4^ Vitamin A/D and vitamin E/selenium injections were producer-reported management practices. Neither exposure was retained in the published final multivariable BRD models, and the article did not report exposure-specific adjusted estimates or confidence intervals.

#### 3.4.1. Controlled Trials

Three controlled trials investigated maternal selenium supplementation [16,53,54] (Table 3 and Supplementary Table S5). Patterson et al. (2013) compared inorganic, organic, and mixed selenium sources administered to late-gestation cows in a research cow-calf pasture setting [16]. No pre-weaning BRD deaths occurred in any treatment group; therefore, the study did not provide an estimable comparative effect of selenium source on BRD mortality. One calf in the mixed-source group died at birth from an event reported separately from BRD mortality [16]. Two other controlled trials reporting secondary outcomes investigated selenium supplementation of beef cows [53,54].

Three CTs reporting BRD-specific outcomes investigated supplementation of beef cows with combinations of trace minerals [14,36,48], and two reported statistically significant differences in a measure of BRD morbidity among offspring [14,36] (Table 3). Grotelueschen et al. (2001) compared three free-choice mineral supplements provided to beef cows during the grazing period, from 4 June to 19–25 September: a control supplement based on salt and dicalcium phosphate; a metal-complex supplement containing additional copper, manganese, zinc, and cobalt; and a sulfate-based inorganic supplement containing the same additional trace minerals [14]. Post-weaning BRD morbidity among offspring did not differ significantly among treatment groups. However, the number of post-weaning “sick incidents,” defined as the combined number of first treatments, retreatments, and repulls, was significantly lower in the metal-complex treatment group (*p* < 0.05) [14].

Marques et al. (2016) compared three supplements provided to pregnant beef cows from 93 to 99 days before calving: a control supplement without additional copper, manganese, zinc, or cobalt; a supplement containing sulfate sources of these minerals; and a supplement containing organically complexed sources of the same minerals [36]. Post-weaning BRD morbidity was assessed during a 45-day preconditioning period, a 112-day growing period, and a feedlot period extending from the end of the growing period to slaughter. No statistically significant differences among treatment groups were detected during the preconditioning or feedlot periods (*p* = 0.95 and *p* = 0.37, respectively). During the growing period, however, BRD morbidity differed significantly among treatment groups and was lower in the group receiving organically complexed minerals (*p* = 0.02) [36]. Neither Grotelueschen et al. (2001) nor Marques et al. (2016) detected statistically significant differences in crude post-weaning mortality among treatment groups [14,36] (Table 3).

Stephenson et al. (2023) compared four supplements provided to beef cows from 91 days before calving to 11 days after calving: a control supplement without additional copper, manganese, or zinc; a supplement containing sulfate sources of these minerals; a supplement containing metal methionine hydroxy analogue sources; and a supplement containing both sulfate and metal methionine hydroxy analogue sources [48]. No statistically significant differences in pre-weaning BRD morbidity among offspring were detected. The authors noted that the low overall morbidity may have limited the ability to detect differences among treatment groups [48].

The creep-feeding study by Wright et al. (2000) [42], described above and summarized in Table 2, compared supplements provided to pre-weaning beef calves with or without additional copper and with varying concentrations of vitamin E and selenium. No statistically significant differences in pre- or post-weaning BRD morbidity were detected among treatment groups [42].

Four additional CTs investigated supplementation of beef cows with copper alone [13] or in combination with manganese and zinc [51,59,66] and reported only secondary outcomes, including crude morbidity or mortality. None of the CTs detected statistically significant differences in crude morbidity or mortality among offspring (*p* > 0.05; Supplementary Table S5). Wilcox et al. (2026) reported no statistically significant difference in pre-weaning calf mortality between treatment groups (3.7% for controls versus 2.2% for treated cows; *p* = 0.54) [66].

Of the four CTs investigating maternal trace mineral supplementation and reporting offspring BRD morbidity or mortality [14,16,36,48], three assessed outcomes in research settings [14,16,48]. Two reported no pre-weaning BRD morbidity [16,48], whereas the third reported a post-weaning BRD incidence of 45% [14]. In the remaining CT, offspring were followed post-weaning in a commercial feedlot. Post-weaning BRD incidence was 34.9% during preconditioning and 42.3% during the growing period, with no cases observed during the subsequent feedlot period [36].

Across the CTs investigating trace mineral supplementation, no estimates of treatment effect magnitude or precision were reported for BRD-specific or secondary health outcomes.

#### 3.4.2. Observational Studies

A survey of 87 western Canadian beef producers found no statistically significant association between injecting beef calves at birth with vitamin E and selenium or vitamin A and pre-weaning treatment for BRD (*p* > 0.20) [19] (Table 3).

An earlier cross-sectional study included 899 calves from 150 western Canadian beef herds surveyed in 2002 [61]. Calves in herds that did not routinely administer vitamin E and selenium at birth had a greater risk of pre-weaning crude mortality than calves in herds that administered these injections (risk ratio = 5.4, 95% CI: 1.85–15.8; *p* = 0.002). The analysis accounted for serum vitamin A concentration quartile, calf age at sampling, serum immunoglobulin G concentration within 1 month after birth, month of calving, cow age at calving, maternal body condition score, assistance at birth, and administration of vitamin E and selenium at birth [61].

### 3.5. Colostrum Management and Passive Transfer

Studies of colostrum management included several designs. Overall, one CT and one cross-sectional study reported BRD-specific outcomes [8,50], and one cross-sectional study reported secondary outcomes only [56]. Studies reporting BRD-specific outcomes are summarized in Table 4, whereas the study reporting secondary outcomes only is summarized in Supplementary Table S6. In addition, eight CTs investigated maternal nutritional supplementation and reported intermediate outcomes related to colostrum or neonatal serum immunoglobulin G (IgG) concentrations [39,43,48,51,52,55,58,60]. Three cross-sectional studies examined associations between neonatal serum IgG concentrations and crude or BRD-specific morbidity or mortality [9,10,11].

**Table 4:** Descriptive summary of colostrum intervention studies reporting BRD-specific outcomes.

Author, Year [Citation]	Intervention or Exposure	Timing (Duration)	Settings	Population- Outcome	Comparator (Incidence of Outcome in Control Group)	Result (*p* Value)
Mitman et al., 2025 [8]	Colostrum intervention timing and volume	Remained with dams as per herd protocol, OR received 167.3 ± 5.9 g IgG within 3 h of birth, OR received 122 ± 6.1 g IgG within 3 h then 123 ± 2.8 g IgG 6–8 h after birth, OR received 83.6 ± 4.1 g IgG within 3 h, 82.8 ± 3.6 g IgG at 6–8 h, and 81.0 ± 3.5 g IgG at 12–14 h after birth	Res cow-calf ^1^ (Res cow-calf)	Pre-weaning BRD morbidity	Natural suckling with the dam	Non-significant (*p* = 0.52) difference across tx gps using Fisher’s exact test
				Pre-weaning BRD mortality	Natural suckling with the dam	Non-significant (*p* = 0.70) difference across tx groups ^2^
Woolums et al., 2013 ^3^ [50]	Bottle or tube used to feed colostrum to more than one calf		Com cow-calf ^1^ (Com cow-calf)	Pre-weaning BRD morbidity	No bottle or tube	*p* < 0.001 association between operations using bottle or tube, and the operation having one or more calves treated for pre-weaning BRD
	More than one calf experiences failure of passive transfer		Com cow-calf (Com cow-calf)	Pre-weaning BRD morbidity	No FPT	*p* < 0.001 association between operations with more than one calf per year experiencing FPT, and the operation having one or more calves treated for pre-weaning BRD

^1^ Res cow-calf = research cow-calf farm, i.e., operated by university or research facility; Com cow-calf = commercial cow-calf farm; Com feedlot = commercial feedlot ^2^ Across tx gp = significance of difference in the outcome is assessed across multiple treatment or control groups as opposed to contrasts. ^3^ Cross-sectional study. Studies cited without superscript were controlled trials.

#### 3.5.1. Studies Reporting BRD-Specific Outcomes

One recent CT compared three colostrum-replacement strategies with natural suckling of the dam as the control [8]. The strategies provided 170 g of immunoglobulin (Ig) at 3 h of age, 250 g of Ig divided between two feedings completed within 6–8 h of age, or 250 g of Ig divided among three feedings completed within 12–14 h of age. No statistically significant differences in pre-weaning BRD morbidity or mortality were detected among treatment groups (*p* > 0.05) [8].

In a cross-sectional survey of 459 beef herds in Florida, Georgia, West Virginia, Iowa, Kansas, and Nebraska, Woolums et al. (2013) found that using the same bottle or tube to feed colostrum to more than one calf was associated with greater odds of the herd reporting treatment of one or more calves for pre-weaning BRD in univariable logistic regression (*p* < 0.001) [50]. Herds reporting failure of passive transfer (FPT) in one or more calves were also more likely to report treating at least one calf for pre-weaning BRD in univariable analysis (*p* < 0.001). However, neither factor remained statistically significant in the final multivariable model [50].

#### 3.5.2. Study Reporting Secondary Outcomes

In a cross-sectional survey of western Canadian beef cow-calf management practices, routine administration of colostrum to calves born following dystocia was associated with a 0.8-percentage-point reduction in crude mortality from 7 days of age to weaning compared with no intervention (*p* = 0.02) [56].

#### 3.5.3. Controlled Trials Reporting Surrogate Outcomes

Eight CTs investigated the effects of maternal nutrition during gestation on colostrum IgG or neonatal serum IgG concentrations in offspring [39,43,48,51,52,55,58,60]. These nutritional interventions were relevant to the review, but the studies assessed surrogate outcomes that occurred temporally between the interventions and the preferred clinical outcomes of BRD morbidity and mortality.

Six of the eight CTs detected no statistically significant differences in colostrum IgG or neonatal serum IgG concentrations among treatment groups (*p* > 0.05) [39,48,51,52,55,60]. Radunz et al. (2012) compared three maternal diets: hay offered ad libitum, limit-fed corn, and dried corn distillers’ grains with solubles (DDGS) [39]. Three studies investigated mineral supplementation during gestation [48,51,55], and another compared different levels of dietary energy [52]. An earlier 3-year study used a factorial design to investigate protein supplementation during the final trimester and grazing on irrigated pasture rather than feeding hay in a dry-lot during the month before the start of the breeding season [60].

In contrast, Ricks (2018) [58] evaluated supplementation of beef cows with rumen-protected unsaturated fatty acids beginning 94 days before calving. Colostrum IgG and neonatal serum IgG concentrations were significantly greater in the supplemented group than in the control group (*p* < 0.05) [58]. Brandão et al. (2020) also reported significantly different colostrum IgG concentrations between cows supplemented during gestation with calcium salts of soybean oil and those receiving prilled fat (*p* = 0.02) [43].

#### 3.5.4. Observational Studies Reporting Surrogate Outcomes

Three cross-sectional studies assessed associations between neonatal serum immunoglobulin G (IgG) concentrations, an indicator of passive transfer status, and crude pre-weaning morbidity and mortality [9,10,11]. Across the three studies, conducted over two decades in Canada and the United States, neonatal serum IgG concentrations below 24 g/L, measured between 24 h and 8 days of age, were consistently associated with an increased risk of pre-weaning morbidity or mortality (*p* < 0.05; Supplementary Table S6) [9,10,11]. However, Waldner and Rosengren (2009) found no statistically significant association between neonatal serum IgG concentrations categorized as failure of passive transfer (FPT) and pre-weaning treatment for pneumonia [11]. Dewell et al. (2006) [10] also found no statistically significant associations between neonatal serum IgG concentrations and post-weaning morbidity, mortality, or average daily gain. The authors presented power calculations indicating that the study had insufficient power to detect differences in these outcomes [10]. Gamsjäger et al. (2023) additionally reported that calves that had received a colostrum intervention had greater odds of pre-weaning treatment in univariable analysis (*p* = 0.02) and greater odds of pre-weaning mortality in every multivariable model, with odds ratios ranging from 6.1 to 12.7 (*p* ≤ 0.011) [9] (Supplementary Table S6).

### 3.6. Cross-Study Design and Reporting Characteristics

In CTs reporting primary or secondary outcomes, investigators generally reported statistical comparisons of health outcomes among treatment groups but did not report comparative effect estimates, such as risk ratios, rate ratios, or risk differences, to characterize the magnitude of intervention effects (Table 2, Table 3 and Table 4 and Supplementary Tables S4–S6). However, all CTs reporting statistically significant differences in health outcomes among treatment groups (*p* < 0.05) identified which treatment group had the more favourable outcome. Two reported a sample-size calculation [60,66]; only one of these [60] targeted a BRD-relevant outcome, the other having been powered to detect a difference in pregnancy rate [66].

Among the CTs reporting BRD-specific or secondary outcomes, 11 identified BRD as an outcome of interest [8,14,15,36,38,39,40,42,43,49,51], and 22 reported random allocation to treatment groups [8,13,15,16,37,38,39,41,44,45,46,47,49,53,54,55,57,58,63,64,65,66]. One reported blinding outcome assessors to intervention status [60]. Ten reported that one or more study animals were deficient in at least one nutrient [8,13,14,39,42,48,53,55,65,66]. Eighteen used one or more feeding procedures, such as Calan gates for individual feed delivery, that are not commonly used in commercial production [13,15,16,36,38,39,40,42,43,45,47,48,49,51,55,57,58,64]. Ten collected repeated observations over two or more production cycles from the same cows or their offspring [15,39,40,44,45,49,53,57,60,66] (Supplementary Table S3). Twelve reported commingling calves from different treatment groups during part or all of the outcome-assessment period [8,14,15,36,40,43,48,51,55,62,63,64], including nine during the post-weaning period [14,15,36,40,43,51,62,63,64] (Table 1 and Supplementary Table S2).

## 4. Discussion

This review identified four principal findings. First, maternal protein supplementation showed the most consistent preliminary signal among the interventions identified, although only five trials evaluated this intervention and two favourable studies arose from the same research program. Second, selected trace mineral interventions improved outcomes during specific post-weaning periods, but benefits were inconsistent across studies or throughout follow-up. Third, creep feeding was not consistently associated with lower BRD occurrence. Fourth, colostrum and neonatal IgG studies supported a biologically plausible relationship between passive immunity and calf health, but most evidence was observational or based on intermediate outcomes rather than direct tests of BRD-prevention strategies. Overall, only 22 studies conducted across more than two decades of North American research reported BRD-specific morbidity or mortality following nutritional management of beef cows or pre-weaning calves. A distinctive feature of this review was the integration of direct BRD outcomes with secondary, surrogate, and tertiary evidence relevant to causal pathways between maternal or calf nutrition and respiratory health, while maintaining clear distinctions between intermediate responses and evidence of clinical BRD prevention.

Maternal protein supplementation was the only intervention for which multiple controlled trials reported favourable post-weaning BRD outcomes, with three of five trials reporting differences among treatment groups [15,38,44]. Two of these three trials were reported by the same research program [15,38]; because they share investigators, study site and methods, they provide fewer independent lines of evidence than the number of trials alone would suggest. The baseline protein status of the cattle in these studies was unclear, although the reported mean BCS scores would preclude marked deficiency. Two of five maternal protein trials followed offspring in commercial feedlots, where BRD occurrence was generally higher than in research feedlots. The greater number of observed events may have increased the opportunity to detect differences among treatment groups, although it does not by itself strengthen causal validity.

Only two of the five protein studies, both from the same research group, reported BRD findings in the abstract [15,38]. It is possible that BRD was not an outcome of primary interest in the other studies. For each study, but especially those for which BRD may not have been a major focus of the researchers, associations identified should be critically assessed for the likelihood of a causal relationship. Several pertinent criteria for this purpose include the strength of the association measured as a ratio or risk difference, consistency across studies, and coherence, meaning that the observed association can be explained by recognized disease mechanisms [76]. Fetal programming was the principal mechanism proposed by the protein-supplementation studies. The Barker hypothesis links adverse prenatal conditions followed by abundant postnatal nutrition with later metabolic disease [77]. Evidence from human research supports associations between nutritional stress in utero and later metabolic disease [78]. The complementary “silver spoon” hypothesis proposes that favourable prenatal conditions may improve later fitness [77]. These concepts provide biological plausibility but do not establish that fetal programming mediated the BRD findings. Future trials should measure prespecified immune or metabolic intermediates together with clinical BRD outcomes, particularly where low-quality forage or drought increases the risk of maternal protein deficiency.

Trace mineral supplementation is a common component of BRD management in U.S. and Canadian feedlots [79]. Recent research indicates the impact of some forms (organically complexed) of trace minerals on adaptive immune system function and carbohydrate and fat-soluble vitamin metabolism in stockers, providing a plausible mechanism of action when the intervention is applied to this group [79]. Additionally, Canadian surveys indicate that mineral inadequacy is relevant in some production settings, identifying 53% of western, and 77% of eastern weaned calves sampled having less than adequate selenium, and 17% of western, and 14% of eastern calves categorized as copper deficient [80]. Deficiency prevalence alone does not demonstrate that routine supplementation prevents BRD. Both recent primary research [81] and systematic reviews [82,83] have reported a non-significant association between trace mineral supplementation of post-weaning calves and BRD morbidity. In this scoping review, three of four captured CTs reporting BRD-specific outcomes investigating gestational trace mineral supplementation and pre- or post-weaning BRD morbidity reported non-significant (*p* > 0.05) associations with supplementation. Only one CT [36] reported a significant association between treatment group and BRD morbidity during the backgrounding period, with aggregated data for the entire post-weaning period not available. Investigators noted that liver biopsies demonstrated that all treatment groups had similar and adequate cobalt, copper, manganese and zinc liver levels at the start of the study [36]. Commingling of treatment groups, which may dilute group differences, create interference through shared exposures, or introduce pen-level clustering, was reported by the other CTs reporting BRD-specific outcomes [14,48]. The two injectable trace mineral trials added by the updated search were also consistent with this pattern, reporting no significant differences in post-weaning morbidity [65] or pre-weaning calf mortality [66]. Thus, trace mineral supplementation has biological plausibility and selected favourable findings, but the available evidence does not show a consistent reduction in BRD following supplementation of cows or pre-weaning calves.

The dataset of evidence regarding the impacts of colostrum management on BRD in calves included a variety of study designs, and four systematic reviews studying aspects of colostrum management and its impacts on calf health were captured by the search [7,34,35,75]. Two systematic reviews employing meta-analysis reported significantly increased risk of pre-weaning crude morbidity for calves experiencing FPT, although the target population included both beef and dairy calves for both of these reviews [7,75]. The former group of investigators reported that the risk of pre-weaning morbidity was increased (RR = 1.91, 95% CI 1.63–2.24) in European calves experiencing FPT. The risk of BRD-specific morbidity was also increased (RR = 1.75, 95% CI 1.50–2.03) in calves experiencing FPT [7]. These observations are supported by three cross-sectional “surrogate” studies captured in this review, all reporting a significant association (*p* < 0.05) between calves having a neonatal (24 h–8 d) serum IgG concentration less than 24 g/L and an increased risk of morbidity or mortality in pre-weaning calves [9,10,11]. This study-specific threshold of 24 g/L is higher than the thresholds used in the included studies to define FPT (approximately 8–10 g/L) or adequate transfer (approximately 16 g/L) [9,10,11]. The researchers reported 19% to 40% of calves across the three studies falling within the category of less than 24 g/L neonatal serum IgG. These findings support passive immunity as a potentially important component of calf health and justify intervention trials in high-risk calves, including twins and calves experiencing dystocia; however, the observational evidence does not establish that improved colostrum delivery [11,56] reduces herd-level BRD morbidity, antimicrobial treatment, or mortality. A corollary of this observation is that the potential health or production benefits of pushing herd-level mean calf neonatal serum IgG to 24 g/L and beyond are currently unclear.

Additionally, in an observational study of calves from six herds, calves that had received a colostrum intervention had greater odds of pre-weaning treatment (*p* = 0.02) and substantially greater odds of pre-weaning mortality, with odds ratios between 6.1 and 12.7 across multivariable models (*p* ≤ 0.011) [9]. This association should not be interpreted as evidence that colostrum intervention is harmful. Colostrum intervention is typically given to calves born after dystocia or otherwise judged to need assistance, so the association is more likely to identify high-risk calves than an effect of the intervention itself; confounding by indication and residual confounding cannot be excluded. Parallel lines of inquiry could help characterize reasons for this. Commercial colostrum products, as well as current best colostrum management guidelines, have been formulated primarily for dairy calves. Beef-specific products and guidelines could better meet the needs of high-risk beef calves. Ongoing communication of recommendations to beef producers is important to support timely intervention of the correct product in the correct amount [84].

Eight CTs studied the impact of gestational nutrition of the dam on colostrum or neonatal calf serum IgG concentration with two studies of fat supplementation reporting a significant association between intervention and serum IgG concentrations [43,58]. While the significant association reported is noteworthy, none of these CTs reported sample-size calculations or treatment-effect estimates with confidence intervals; this limited assessment of the potential treatment impact.

### 4.1. Review Findings Across Topics

Overall, of the 18 CTs reporting BRD-specific outcomes for nutritional interventions, four reported one or more significant (*p* < 0.05) associations between the nutritional intervention studied and BRD in beef calves. Three of these investigations studied protein supplementation [15,38,44] and one trace mineral supplementation of pregnant cows [36]. Of the 15 CTs studying nutritional interventions but reporting only crude morbidity or mortality outcomes [13,51,52,53,54,55,57,58,59,60,62,63,64,65,66], only one [57] reported a statistically significant health outcome, with increased mortality in the offspring of the control group of cows not receiving fat supplementation. A recent scoping review of risk factors and methods for the prevention of BRD applicable to cow–calf operations similarly reported that of the CTs captured investigating nutritional interventions (n = 47; in beef or dairy animals, and including feedlot applications) 30% reported at least one statistically significant association between intervention and health outcomes. This descriptive count does not account for study size, precision, or selective outcome reporting [73].

The CTs captured in this review did not report pairwise treatment effects and their precision, for BRD-specific or secondary health outcomes. Counts of studies reporting only statistical significance (*p* < 0.05) across treatments do not measure comparative efficacy because study size, precision, outcome definitions, and independence of research groups varied. The design characteristics of the included studies are therefore central to interpretation. Of the 22 studies reporting BRD-specific outcomes, only half included BRD outcomes in the title or abstract. BRD outcomes were therefore likely not the major focus for most of the 18 CTs reporting BRD-specific outcomes, although they were reported in text or tables. These studies may not have been designed to maximize capacity for detection of BRD-associated treatment effects.

Several factors may have contributed to some studies reporting relatively low BRD incidence, which could limit study capacity to detect an effect. Some settings tended to have lower overall rates of BRD, which could have impacted study potential to detect significant treatment effects. While interventions applied to cows or pre-weaning calves were consistently applied on research cow-calf farms affiliated with a university or research-based organization, post-weaning health assessments of BRD morbidity and mortality were performed in research (n = 10 studies) and commercial (n = 5) feedlots. The research feedlots tended to have lower incidence of post-weaning BRD relative to the commercial ones, which could make detection of a difference in BRD across treatment groups more difficult. Additionally, for both pre- and post-weaning BRD morbidity or mortality outcomes, researchers reported assessment periods other than the entire pre- or post-weaning period. Shortened periods of observation for BRD could result in lower BRD incidence being reported, relative to the entire pre- or post-weaning period.

### 4.2. Other Study Design Characteristics Impacting Findings

Twelve of the CTs reporting BRD-specific or secondary outcomes commingled post-weaning calves across intervention groups. Depending on timing and pen allocation, commingling may dilute group differences, create interference through shared exposures, or introduce clustering that should be addressed in the analysis.

One-third of CTs reporting BRD-specific or secondary outcomes used repeated measurements over multiple calving seasons on the same group of study cows. Obtaining additional samples by studying cows in successive years could be considered useful by researchers for increasing the total sample size and the power to detect a treatment effect. However, observations derived from one cow or her offspring over several years cannot be assumed to be independent. Failure to account for repeated observations from the same dams or herds may produce incorrect standard errors and confidence intervals; positive within-cluster correlation commonly leads to underestimated uncertainty when observations are analyzed as independent [85]. Only two of the captured studies reporting repeated measures also reported adjusting for this; one reported zero incidence of BRD morbidity or mortality [45], and the other reported no BRD-specific outcome [66]. For the other studies using repeated measures but not reporting statistical adjustment for this, the reported uncertainty may be inaccurate, and statistical significance may be overstated.

The captured interventions involved several dimensions of complexity as defined by Cochrane. Some had multiple components, as well as the potential for interaction or effect modification with other elements of the production system such as other ration ingredients or pasture quality. The study populations in some investigations included individuals with differing baseline levels of the nutrients being studied, which could increase variation in clinical treatment response. Some studies conducted over the grazing period described drought during this time, with both the impact of the drought itself on forage quality and quantity, as well as investigators’ response (e.g., offering additional supplements) potentially affecting findings [14].

One of the greatest challenges in assessing and interpreting the impacts of nutritional interventions on BRD morbidity or mortality in this review reflects the lack of reporting of sampling strategy and study power in CTs. Basic study design involves defining the acceptable level of type I (alpha) error, i.e., detecting a difference between treatment groups when none truly exists [86]. This is typically set at a value of 0.05 and is consistently reported in captured studies in this review. In contrast, the definition of acceptable level of type II (beta) error, i.e., failure to detect a treatment effect when one truly exists, which will then drive calculations of required sample size to detect the hypothesized treatment effect, was rarely reported. The near absence of reported sample size calculations in captured studies suggests the potential for these studies to lack sufficient power to detect a BRD treatment effect. In livestock field trials, feasible sample size and replication may be constrained by the number of available animals, pastures or pens, study duration, and cost. When the calculated target cannot be achieved, investigators should report the assumptions, practical constraints, and resulting detectable effect or precision. Alternatively, investigators could have shared the non-significant treatment effects with confidence intervals to provide more context to their findings, but these were not reported in the captured CTs. Consequently, the studies reporting a non-significant association between interventions and BRD outcomes cannot be interpreted as evidence that these interventions are truly not effective; the power of these studies to detect a BRD treatment effect is unclear. Given the potential for captured studies to be underpowered for the outcomes of interest to this review due to various combinations of sample size, commingling, low frequency of BRD in some settings, etc., currently the reader is unable to distinguish between no evidence of a treatment effect and evidence of no treatment effect for these studies.

The CTs that reported a significant association between intervention and BRD outcomes also pose some challenges in interpretation. The pertinent results are typically reported as percentages of BRD morbidity or mortality in each treatment group (often with more than two groups per study), with the probability of a difference across groups presented as a *p* value or indicated by superscripts. Reporting treatment effects with measures of magnitude and precision is necessary for cost–benefit assessment.

While investigation of study quality, or risk of bias, was not undertaken, we noted that randomization of intervention allocation was not reported consistently in the captured CTs. In contrast, blocking of groups to achieve equal allocation of known factors which could affect study outcomes such as calf performance (e.g., calving date, or age or weight of dam) was widely practiced. The lack of reported randomization across studies means that findings could be influenced by unknown factors. Blinding of health assessors to intervention status, which is important to reduce detection bias, was rarely reported. Blinding is employed to prevent outcome assessors (for our research question, health assessors) from more frequently assessing disease in a specific treatment group, such as the control group, due to their underlying bias. The low frequency of reported blinding could reflect challenges due to study logistics such as limited number of pens available. Overall, lack of reported randomization or blinding in some studies increases the potential for selection or detection bias to impact findings. Similar observations were made by Chen et al. (2022) regarding that dataset of captured CTs [73].

The observational studies also had some potential sources of bias, including selection and reporting bias. It is possible that those producers consenting to participate differed from the target population of beef farmers, for example, in their level of interest in animal health. Similarly, participants could be affected by reporting bias and might be more likely to report successful or positive experiences, or those outcomes perceived to be more favourably viewed by researchers.

One form of bias potentially applicable to both CTs and observational studies is misclassification, particularly as it pertains to considering crude morbidity as an indicator of BRD-specific morbidity. A recent western Canadian analysis of 2024 morbidity data from 162,910 Alberta cattle from 531 closed production lots in 21 feedlots reported morbidity due to respiratory disease (BRD and atypical interstitial pneumonia), metabolic disease (gas bloat, frothy bloat, grain overload and caudal vena cava thrombosis) and lameness (foot rot, toe abscesses, arthritis, digital dermatitis and laminitis) [87]. While respiratory disease was consistently the major cause of morbidity, the relative proportion attributable to the three major causes varied with the season of arrival at the feedlot, with more than 25% of fall-placed calves experiencing respiratory morbidity, 5% lameness, and 0.59% metabolic disease [87]. In winter-placed calves, the first pull rate was between 10% and 15% for both respiratory disease and lameness. Therefore, the degree of misclassification involved in using post-weaning morbidity as an indicator of post-weaning BRD morbidity could vary with the specific feedlots involved, as well as the season of arrival of the cattle sampled. As described above, the studies set in research feedlots may have been biased towards the null for BRD morbidity or mortality assessments given the generally lower incidence in research settings, with a consequent possible reduction in capacity for detecting a treatment effect.

Across the 22 studies categorized in this review as reporting BRD outcomes, specific clinical outcomes reported included BRD, treatment for BRD, or treatment for respiratory disease. While a Canadian cow-calf survey reported similar proportions of pre-weaning respiratory morbidity relative to treatment [19], it is possible that the use of different metrics across studies could reduce the comparability of specific studies.

Overall, this review identified some opportunities for study design, execution, and analysis of prospective studies of nutrition on BRD outcomes, to enhance fitness for purpose. Involving investigators with a range of expertise, including nutritionists and veterinary practitioners, at the planning stage would help develop a design most suitable for the study question, capable of measuring the preferred health outcomes and detecting a treatment effect with desired precision. Preferred clinical outcome measures would necessarily be BRD-specific rather than crude measures of morbidity or mortality, as these are not interchangeable. If the study involves the post-weaning period, a commercial feedlot setting could yield a higher baseline level of BRD cases, possibly improving chances of detecting a true treatment effect, relative to a research setting.

Randomization of treatment allocation is useful even in studies planning on blocking subjects by effect modifiers, particularly to mitigate the impacts of unknown sources of bias or confounding. Blinding of outcome assessors to treatment status may not be feasible, especially in trials with small numbers of subjects, but if possible, reduces the potential for bias in identifying BRD. Study power assumptions and calculations are important to the reader to support their assessment of results. Presentation of treatment effect magnitude and precision are also important for understanding results as well as their extrapolation for other purposes such as cost–benefit estimates. Since commingling of treatment groups may restrict health assessments to direct effects, avoiding commingling, where feasible, is desirable.

A sequential approach of CTs studying targeted specific interventions such as gestational protein supplements, followed by observational studies capturing herd-level treatment effects in a variety of contexts, could improve understanding of intervention treatment effects in North American commercial beef herds. The existing published studies were frequently conducted in research settings, especially at the pre-weaning stage, and many involved feeding procedures not typically employed in commercial operations. Replication of studies reporting a beneficial treatment effect, such as protein, fat, or trace mineral supplementation, employing commercially used methods for supplement delivery, could be useful.

Intervention complexity was considered during data extraction and narrative synthesis rather than treated solely as a reporting characteristic. Differences in intervention components, timing, delivery, baseline nutritional status, and production context informed the grouping and interpretation of studies and contributed to the decision not to combine results quantitatively [29,30]. This framework helps explain the heterogeneity of findings and why a single nutritional recommendation cannot be inferred from this evidence map. An intervention that is effective in nutrient-deficient cattle or during drought may not have the same effect in adequately nourished cattle or under different management conditions. Future studies should therefore describe intervention components and context explicitly, prespecify plausible effect modifiers and mediators, and evaluate commercially feasible strategies in the settings for which recommendations are intended.

Our study has several limitations. A formal study-level risk-of-bias assessment was not performed, which limits confidence in causal interpretation and prevents ranking interventions by certainty of evidence. A future systematic review with structured risk-of-bias assessment could support more specific recommendations. The rapid-review approach relied on one reviewer to screen the remaining citations after duplicate assessment of a 50-citation calibration sample, which may have increased the risk of missed eligible studies or screening errors. The search was restricted to North American studies published from 2000 onward in English or French, and studies assessing outcomes only during the first seven days of life were excluded, a restriction particularly relevant to interpretation of the colostrum-management evidence. Although studies reporting tertiary outcomes only were mapped for completeness, detailed extraction and synthesis focused on the primary and secondary outcomes most relevant to the review question. The direction and magnitude of any effects of these review limitations on the findings could not be determined.

## 5. Conclusions

This rapid scoping review identified a growing body of evidence that nutritional management of beef cows may influence BRD risk in their offspring. One of the most consistent preliminary signals reported was for protein supplementation of pregnant cows, although 2 of the 3 studies reporting favourable findings originated from the same research group. Selected studies also identified potential benefits from trace mineral and fat supplementation. Evidence concerning colostrum management supported biological plausibility but did not establish a consistent reduction in BRD. These findings support further evaluation of nutritional strategies as components of integrated BRD prevention programs. However, differences in interventions, production settings, outcome definitions, and statistical reporting limit the formulation of specific recommendations; the observed preliminary signals should not be interpreted as established comparative efficacy. Future studies should prioritize interventions with favourable preliminary evidence, use adequately powered designs in commercial production settings, measure prespecified BRD-specific outcomes, and report effect sizes with confidence intervals. Strengthening this evidence base could support practical nutritional recommendations that improve calf health, animal welfare, production efficiency, and antimicrobial stewardship.

## Figures and Tables

**Table 1:** Descriptive summary of 22 included studies reporting one or more BRD-specific outcomes.

Author, Year [Citation]	Location	Intervention or Exposure	Population Sampled for Intervention (Outcome Assessment)	Total Number Studied for Intervention (Outcome Assessment)	Replicates for Intervention (Outcome Assessment)	Clinical Outcomes Assessed (Post-Weaning Study Period)
Arthington et al., 2008 [37]	US (Florida)	Creep feeding supplement 15.1% crude protein (CP), 1:1 Ca:P	Pre-weaning calves (Post-weaning steers)	Year 1 = 64 calves, Y2 = 32	NR (4 each of 4 treatment (tx) group (gp)s; 4 and 2 steers/pen Years 1 and 2 respectively)	BRD morbidity (Post-weaning study period = 29 d)
Brandão et al., 2020 [43]	US (Oregon)	Ca salts of soybean oil or prilled saturated fat	Cows (Post-weaning calves)	104 cows	Two pasture replicates, each containing 26 cows per treatment (Offspring commingled during feedlot assessment)	BRD morbidity (To slaughter)
Grotelueschen et al., 2001 [14]	US (Nebraska)	Inorganic or organic Cu, Mn, Zn, Co supplement (sup), negative control	Cows (Post-weaning calves)	641 cows	3 pastures for each of 3 tx gps (3 commercial pens containing 618 calves in total, commingled)	BRD morbidity and mortality (To slaughter)
Hanzlicek et al., 2013 ^1^ [18]	US ^2^	Creep feeding	Pre-weaning calves (Pre-weaning calves)	443 herds	NA	BRD morbidity NA
Larson et al., 2009 [44]	US (Nebraska)	Basal diet winter range-corn residue ± 28% protein sup; Factorial design	Cows (Pre- and post-weaning steers)	Y1 = 109 cows, 51 steers; Y2 = 114 cows, 58 steers; Y3 = 116 cows, 63 steers	2 reps each of 4 tx gps each of 3 yr (2 pens per tx gp)	BRD morbidity (To slaughter)
Marques et al., 2016 [36]	US (Oregon)	Inorganic or organic Cu, Mn, Zn, Co supplement, negative control	Cows (Pre- and post-weaning calves)	84 cows	7 reps for each of 3 tx gps each containing 4 cows (One commingled replicate)	BRD morbidity, (To slaughter; Preconditioning (45 d), growing (112 d), and finishing periods reported separately)
Mitman et al., 2025 [8]	US (North Carolina)	3 protocols for timing of colostrum delivery, plus normal suckling	Neonatal calves (Pre-weaning calves)	60 calves	2 replicates total with tx arms commingled also with non-study cattle	BRD morbidity, BRD mortality, (NA)
Moriel et al., 2020 [45]	US (Florida)	Sugarcane molasses and urea + methionine hydroxy analog sup	Breeding heifers (Pre- and post-weaning calves)	72 heifers (hrfs) (4 steer and 4 heifer calves randomly selected, per tx)	4 pastures/tx/yr, for 2 tx gps each, over 2 years (Early-weaned at 89 ± 5 d old, fed individually)	BRD morbidity, BRD mortality (Early weaned at 89 d old; studied for an additional 54 d)
Mulliniks et al., 2013 [38]	US (New Mexico)	36% CP cottonseed meal + urea sup, or 28% CP corn meal, dried distillers’ grains (DDG) plus trace mineral sup, negative control	Cows (Post-weaning steers)	103 cows (62 steers)	2 replicates per tx gp for each of 3 tx (All 62 steers fed as one gp)	BRD morbidity and mortality (To slaughter)
Mulliniks et al., 2012 [15]	US (New Mexico)	36% cottonseed meal (CSM) hand-fed; self-fed blood and feather +TM sup; brief intermittent CSM offered as required	Cows (Post-weaning steers)	333 cows total (103 steers)	2 pastures/tx/yr for 4 years; cows randomly assigned to potentially different tx each year (Steers (n = 103) commingled in one gp)	BRD morbidity and mortality (To slaughter)
Palmer et al., 2021 [46]	US (Arkansas)	Creep with or without yeast	Pre-weaning calves (Pre- or post-weaning calves)	95 pairs with heifer calves	5 pasture replicates for each of 2 tx gp (5 pasture reps per tx arm)	BRD morbidity, BRD mortality (42 d backgrounding on pasture)
Patterson et al., 2013 [16]	US (Kentucky)	Organic, inorganic, or a 1:1 blend of organic and inorganic selenium in a free-choice mineral mix	Late-gestation cows (Pre-weaning calves)	24 cows	1 replicate of 8 cows individually fed, for each tx gp (1 replicate per tx gp)	Pre-weaning BRD mortality, birth through weaning
Radunz et al., 2012 [39]	US (Ohio)	Energy sources from hay, corn or dried distillers’ grains with solubles (DDGS)	Cows (Pre- and post-weaning calves)	Y1 = 144 cows (25 hfrs, 21 bulls fed HY, 18 hfrs, 20 bulls fed CN, 18 hfrs, 24 bulls fed DDGS) Y2 = 84 cows (17 hfrs, 6 bulls fed HY, 16 hfrs, 9 bulls fed CN, 12 hfrs, 13 bulls fed DDGS)	Y1 = 9 reps; Y2 = 12 reps (Feedlot: Individual pens)	BRD morbidity and mortality (To slaughter; 28 d backgrounding + individual finishing)
Schubach et al., 2019 [47]	US (Oregon)	Creep with Ca salts of soybean oil (CSSO) or prilled fat	Pre-weaning calves (Post-weaning steers)	Y1 = 32 steers Y2 = 32	4 replicates per tx (4 replicates per tx)	BRD morbidity (Creep feeding, Growing, and Feedlot arrival to slaughter)
Shike et al., 2007 [41]	US (Illinois)	Creep with or without fibre, or no creep (additionally also early weaning)	Pre-weaning calves (Pre- and post-weaning steers)	168 total across 4 tx gps	6 reps per tx gp; (NR)	BRD morbidity, BRD mortality (To slaughter; 331–334 d)
Shoup et al., 2015 [40]	US (Illinois)	2 levels (low or high) of energy-protein sup	Cows (Post-weaning steers)	Y1 = 326 cows (134 steers) Y2 = 383 (147 steers)	3 reps per tx; (6 reps commingled)	BRD morbidity and mortality
Stephenson et al., 2023 [48]	US (Missouri)	Inorganic, organic or combination of forms of Cu, Mn, Zn, negative control	Cows (Pre-weaning calves)	48 cows	4 cows per pen, all tx gps in each pen; postcalving one gp commingled	BRD morbidity (NA)
Tjardes, 2020 [49]	US (Iowa)	Creep feed, negative control	Pre-weaning calves (Post-weaning calves)	Y1 = 103 calves Y2 = 100	2 reps of 25 cows per tx; (Y1 4 reps per tx; Y2 2 reps per tx; not commingled)	BRD morbidity and mortality (To slaughter)
Waldner et al., 2022 ^1^ [19]	Canada (Alberta, Saskatchewan, Manitoba)	Creep feeding (yes/no); vitamin A/D injection near birth (yes/no); vitamin E/ selenium injection near birth (yes/no)	Pre-weaning calves in commercial cow-calf herds (Pre-weaning calves)	87 herds	NA	Proportion of pre-weaning calves treated for BRD
Woolums et al., 2018 ^1^ [20]	US (Nebraska, North Dakota, South Dakota)	Supplements fed to cows (Y/N)	Cows (Pre-weaning calves)	30 case + 54 control herds	NA	BRD morbidity (NA)
Woolums et al., 2013 ^1^ [50]	US (Florida, Georgia, West Virginia, Iowa, Kansas, Nebraska)	Creep feeding (Y/N) Bottle or tube used to feed colostrum to >1 calf (Y/N)	Pre-weaning calves (Pre-weaning calves)	459 herds	NA	BRD morbidity (NA)
Wright et al., 2000 [42]	US (Kansas)	Creep with Cu or Cu + Se, negative control	Pre-weaning calves (Post-weaning calves)	151 calves	1 rep for each tx gp; daily calves sorted to tx gp and individually fed for 49 d expt 1, 53 d expt 2	BRD morbidity Rectal temperatures (28 d receiving diet reported expt 1; 43 d backgrounding ration expt 2)

Abbreviations: Ca = Calcium; Co = Cobalt; Cu = Copper; Expt = experiment; Gp = group; Ig = Immunoglobulin; Mn = Manganese; NR = not reported; P = Phosphorus; rep = replicate; Se = Selenium; tx = treatment; Y/N = yes/no; Y1 = Year 1; Zn = Zinc. ^1^ Study design: Cross-sectional, except for Woolums et al., 2018 [20] (case–control design). All citations not superscripted reflect controlled trials. ^2^ Twenty-four states were sampled: Alabama, Arkansas, California, Colorado, Florida, Georgia, Idaho, Iowa, Kansas, Kentucky, Louisiana, Mississippi, Missouri, Montana, Nebraska, New Mexico, North Dakota, Oklahoma, Oregon, South Dakota, Tennessee, Texas, Virginia, and Wyoming. States were chosen on the basis of the stated goal that the survey would represent at least 70% of the beef cows and cow-calf producers in the United States.

## Data Availability

All data generated during this review are contained within the article and its Supplementary Materials. The review protocol is available in the University of Saskatchewan HARVEST repository at the persistent link provided in the Introduction.

## Supplementary Materials

The following supporting information can be downloaded at: https://www.mdpi.com/article/10.3390/ani16193116/s1, Supplementary File S1a, Search Strategy; Supplementary File S1b, Level 1 Relevance Screening Form; Supplementary File S1c, Level 2 Relevance Confirmation Form; Supplementary File S1d, Data Extraction Form; Supplementary File S1e, Completed PRISMA-ScR Checklist; Supplementary Table S2, Studies Reporting Secondary or Tertiary Outcomes Only; Supplementary Table S3, Additional Characteristics of Included Studies; Supplementary Table S4, Macronutrient Interventions Reporting Secondary Outcomes; Supplementary Table S5, Trace Mineral and Vitamin Interventions Reporting Secondary Outcomes; Supplementary Table S6, Colostrum Management, Passive Transfer, and Immunoglobulin Outcomes.
