# Supplementary material for: A Rapid Scoping Review of Nutritional Management of Beef Cows and Pre-Weaning Calves and the Occurrence of Bovine Respiratory Disease in Pre- and Post-Weaning Calves in North America
Source: Animals (Basel). 2026 Oct 4;16(19):3116. doi: 10.3390/ani16193116 (PMC13663391; doi:10.3390/ani16193116)
Supplement: Supplementary file 1 [file animals-16-03116-s001.zip › Supplementary_Files_S1a-S1e.pdf]

Supplementary File S1a. Search Strategy

Search dates: 7 October 2025; updated 30 August 2026.

Databases searched: AGRICOLA, MEDLINE, PubMed, Scopus, Web of Science, and ProQuest Dissertations and Theses.

The searches combined terms for beef-cattle populations, nutritional or colostrum interventions, and respiratory or broader health outcomes. Searches were limited to publications from 2000 onward where the database interface permitted. North American eligibility was confirmed during screening.

Records exported before deduplication

Database	Records exported 2025	Records exported 2026
PubMed	3,152	293
Web of Science	2,270	917
Scopus	1,194	421
AGRICOLA	883	62
MEDLINE	815	204
ProQuest Dissertations and Theses	1,124	103
Total	9,438	2,000

Combined across the original and updated searches, the records exported before deduplication were PubMed 3,445; Web of Science 3,187; Scopus 1,615; AGRICOLA 945; MEDLINE 1,019; and ProQuest Dissertations and Theses 1,227, giving the total of 11,438 shown in Figure 1.

Other identification methods

An additional 27 records were identified through other methods: 14 through forward or backward citation searching and 13 through grey-literature searching. These records were tracked separately from the 11,438 records exported from bibliographic databases.

Search concepts

Population terms: cow-calf OR pair* OR cow OR cows OR calf OR calves OR “beef cattle” OR cattle OR steer OR steers OR heifer OR heifers OR stocker* OR feeder*

Nutrition and management terms: nutrition OR “body condition score” OR BCS OR “fetal programming” OR “energy supplementation” OR “protein supplementation” OR “maternal nutrition” OR “offspring development” OR “developmental programming” OR vitamin* OR mineral* OR water OR Vitaferst-Care OR Multimin OR survey OR management

Colostrum and passive-transfer terms: colostrum OR colostral OR “colostrum management” OR “failure of passive transfer immunity” OR FPTI OR “failure of passive transfer” OR FPT OR survey OR management

Creep-feeding terms: creep OR “creep feeding” OR “calf supplement” OR “calf supplementation”
Outcome terms: BRD OR “bovine respiratory disease” OR pneumonia OR “shipping fever” OR morbidity OR mortality OR illness OR death OR pull* OR treatment

Recorded search combinations

Beef AND (population terms) AND (nutrition and management terms) AND (outcome terms)
Beef AND (population terms) AND (colostrum and passive-transfer terms) AND (outcome terms)
Beef AND (population terms) AND (creep-feeding terms) AND (outcome terms)

Database documentation 7 Oct. 2025

Database	Fields or tags recorded	Search date	Limits recorded	Records exported
AGRICOLA	Keyword search; exact final field tags not recorded	7 October 2025	English or French; 2000 onward, where permitted	883
MEDLINE	Keywords with expanded subject headings	7 October 2025	English or French; 2000 onward, where permitted	815
PubMed	Title, abstract, and keyword terms recorded	7 October 2025	English or French; 2000 onward, where permitted	3,152
Scopus	Title, abstract, and keywords	7 October 2025	English or French; 2000 onward, where permitted	1,194
Web of Science	ALL field	7 October 2025	English or French; 2000 onward, where permitted	2,270
ProQuest Dissertations and Theses	NOFT field	7 October 2025	English or French; 2000 onward, where permitted	1,124

Citation searching

Backward reference-list searching and forward citation searching were conducted for relevant reports and key papers.

Grey-literature searching

The Bovine Practitioner, 2000 to the search date

American Association of Bovine Practitioners annual conference publications, 2000 to the search date
Academy of Veterinary Consultants meeting publications, 2000 to the search date

Bovine Respiratory Disease Symposium publications, 2000 to the search date
Google searches using the electronic search concepts

Detailed database search strings and staged retrieval counts

The following table reproduces the database-specific strings, field conventions, staged hit counts, and standalone hit counts documented on 7 October 2025 in the original search record. Q1 addressed nutrition and management, Q2 addressed colostrum and passive transfer, and Q3 addressed creep feeding. Counts shown within the staged strings are search-development counts; the final exported totals used in the review flow diagram are reported above.

Note: ALL indicates the Web of Science All Fields search; TITLE-ABS-KEY indicates Scopus title, abstract, and keyword searching; and NOFT indicates the ProQuest field searching anywhere except full text, as recorded in the original search documentation.

Database	String	Hits as part of string (Standalone hits)
Agricola		
Q1	Beef AND	66,375
	(cow-calf OR pairs OR cows OR calves OR “beef cattle” OR beef OR	42,367
	cattle OR cow OR cows OR steer OR steers OR heifer OR calf OR calves OR stocker* OR feeder*) AND	(435,737)
	(nutrition OR "body condition score" OR BCS OR "fetal programming" OR "energy supplementation" OR "protein supplementation" OR "maternal nutrition" OR "offspring development" OR "developmental programming" OR vitamin* OR mineral* OR water OR creep OR Vitaferst-Care OR Multimin OR survey OR management) AND	11,994 (2,392,623)
	(BRD OR "bovine respiratory disease" OR pneumonia OR "shipping fever" OR morbidity OR mortality OR illness OR death OR pulls OR treatment)	421 (272,550)
Q2	Beef AND	66,375
	(cow-calf OR pairs OR cows OR calves OR “beef cattle” OR beef OR cattle OR cow OR cows OR steer OR steers OR heifer OR calf OR calves OR stocker* OR feeder*) AND	42,367 (435,737)
	(colostrum OR colostral OR "colostrum management" OR "failure of passive transfer immunity" OR FPTI OR "failure of passive transfer" OR FPT OR survey OR management) AND	5,312 (952,143)
	(BRD OR "bovine respiratory disease" OR pneumonia OR "shipping fever" OR morbidity OR mortality OR illness OR death OR pulls OR treatment)	300 (272,550)
Q3	Beef AND	66,375
	(cow-calf OR pairs OR cows OR calves OR “beef cattle” OR beef OR cattle OR cow OR cows OR steer OR steers OR heifer OR calf OR calves OR stocker* OR feeder*) AND	42,367 (435,737)
	(creep OR "creep feeding" OR "calf supplement" OR “calf supplementation") AND	148 (4,045)

**Database**	**String**	**Hits as part of string (Standalone hits)**
	(BRD OR "bovine respiratory disease" OR pneumonia OR "shipping fever" OR morbidity OR mortality OR illness OR death OR pulls OR treatment)	3 (272,550)
Web of Science		
Q1	(Beef) AND	93,121
	All=(cow-calf OR pairs OR cows OR calves OR "beef cattle" OR beef OR cattle OR cow OR cows OR steer OR steers OR heifer OR calf OR calves OR stocker* OR feeder*) AND	47,311<br>(1,771,260)
	All=(nutrition OR "body condition score" OR BCS OR "fetal programming" OR "energy supplementation" OR "protein supplementation" OR "maternal nutrition" OR "offspring development"<br>OR "developmental programming" OR vitamin* OR mineral* OR water	17,573<br>(11,158,417)
	OR Vitaferst-Care OR Multimin OR survey OR management) AND	
	ALL=(BRD OR "bovine respiratory disease" OR pneumonia OR "shipping fever" OR morbidity OR mortality OR illness OR death OR pulls OR treatment)	1,196<br>(3,636,319)
Q2	ALL=(Beef) AND	93,121
	ALL=(cow-calf OR pairs OR cows OR calves OR "beef cattle" OR beef<br>OR cattle OR cow OR cows OR steer OR steers OR heifer OR calf OR calves OR stocker* OR feeder*) AND	47,311<br>(1,771,260)
	ALL=(colostrum OR colostral OR "colostrum management" OR "failure<br>of passive transfer immunity" OR FPTI OR "failure of passive transfer"<br>OR FPT OR survey OR management)	8,471<br>(8,303,646)
	ALL= (BRD OR "bovine respiratory disease" OR pneumonia OR "shipping fever" OR morbidity OR mortality OR illness OR death OR	892<br>(3,636,319)

Database	String	Hits as part of string (Standalone hits)
	pulls OR treatment)	
Q3	Beef AND	93,121
	ALL=(cow-calf OR pairs OR cows OR calves OR “beef cattle” OR beef OR cattle OR cow OR cows OR steer OR steers OR heifer OR calf OR calves OR stocker* OR feeder*) AND	47,311 (1,771,260)
	All=(creep OR "creep feeding" OR "calf supplement" OR “calf supplementation")	182 (120,380)
	All=(BRD OR "bovine respiratory disease" OR pneumonia OR "shipping fever" OR morbidity OR mortality OR illness OR death OR pulls OR treatment)	(3,636,319)
Medline	Keywords, expand subject headings	
Q1	Beef AND	
	(cow-calf OR pairs OR cows OR calves OR “beef cattle” OR beef OR cattle OR cow OR cows OR steer OR steers OR heifer OR calf OR calves OR stocker* OR feeder*) AND	25864 (707057)
	(nutrition OR "body condition score" OR BCS OR "fetal programming" OR "energy supplementation" OR "protein supplementation" OR "maternal nutrition" OR "offspring development" OR "developmental programming" OR vitamin* OR mineral* OR water OR Vitaferst-Care OR Multimin OR survey OR management) AND	5931 (4620335)
	(BRD OR "bovine respiratory disease" OR pneumonia OR "shipping fever" OR morbidity OR mortality OR illness OR death OR pulls OR treatment)	432 (3297408)
Q2	Beef AND	
	(cow-calf OR pairs OR cows OR calves OR “beef cattle” OR beef OR cattle OR cow OR cows OR steer OR steers OR heifer OR calf OR	25864 (707057)

	calves OR stocker* OR feeder*) AND	
	(colostrum OR colostral OR "colostrum management" OR "failure of passive transfer immunity" OR FPTI OR "failure of passive transfer" OR FPT OR survey OR management) AND	2538 (2658701)
	(BRD OR "bovine respiratory disease" OR pneumonia OR "shipping fever" OR morbidity OR mortality OR illness OR death OR pulls OR treatment)	327 (3297408)
Q3	Beef AND	
	(cow-calf OR pairs OR cows OR calves OR “beef cattle” OR beef OR cattle OR cow OR cows OR steer OR steers OR heifer OR calf OR calves OR stocker* OR feeder*) AND	25864 (707057)
	(creep OR "creep feeding" OR "calf supplement" OR “calf supplementation") AND	56 (6473)
	(BRD OR "bovine respiratory disease" OR pneumonia OR "shipping fever" OR morbidity OR mortality OR illness OR death OR pulls OR treatment)	0 (3297408)
Scopus	Title abstract keywords	
Q1	Beef AND	72,951
	(cow-calf OR pairs OR cows OR calves OR “beef cattle” OR beef OR cattle OR cow OR cows OR steer OR steers OR heifer OR calf OR calves OR stocker* OR feeder*) AND	26,321 (281,257)
	(nutrition OR "body condition score" OR BCS OR "fetal programming" OR "energy supplementation" OR "protein supplementation" OR "maternal nutrition" OR "offspring development" OR "developmental programming" OR vitamin* OR mineral* OR water OR Vitaferst-Care OR Multimin OR survey OR management) AND	8,523 (15,447,437)
	(BRD OR "bovine respiratory disease" OR pneumonia OR "shipping fever" OR morbidity OR mortality OR illness OR death OR pulls OR treatment)	608 (4,979,895)
Q2	Beef AND	72,951
	(cow-calf OR pairs OR cows OR calves OR “beef cattle” OR beef OR cattle OR cow OR cows OR steer OR steers OR heifer OR calf OR calves OR stocker* OR feeder*) AND	26,321 (281,257)

Finalized 24 August 2026 | PRISMA-ScR checklist

	(colostrum OR colostral OR "colostrum management" OR "failure of passive transfer immunity" OR FPTI OR "failure of passive transfer" OR FPT OR survey OR management) AND	4,734<br>(9,050,633)
	(BRD OR "bovine respiratory disease" OR pneumonia OR "shipping fever" OR morbidity OR mortality OR illness OR death OR pulls OR treatment)	473<br>(4,979,895)
Q3	Beef AND	72,951
	(cow-calf OR pairs OR cows OR calves OR "beef cattle" OR beef OR	26,321
	cattle OR cow OR cows OR steer OR steers OR heifer OR calf OR calves OR stocker* OR feeder*) AND	(281,257)
	(creep OR "creep feeding" OR "calf supplement" OR "calf supplementation")	113 (131,400)
	(BRD OR "bovine respiratory disease" OR pneumonia OR "shipping fever" OR morbidity OR mortality OR illness OR death OR pulls OR treatment)	(4,979,895)
Pubmed	Title abstract keywords	
Q1	Beef AND	46,213
	(cow-calf OR pairs OR cows OR calves OR "beef cattle" OR beef OR cattle OR cow OR cows OR steer OR steers OR heifer OR calf OR calves OR stocker* OR feeder*) AND	27,754<br>(526,697)
	(nutrition OR "body condition score" OR BCS OR "fetal programming" OR "energy supplementation" OR "protein supplementation" OR "maternal nutrition" OR "offspring development" OR "developmental programming" OR vitamin* OR mineral* OR water OR Vitaferst-Care OR Multimin OR survey OR management) AND	9,720<br>(8,116,492)
	(BRD OR "bovine respiratory disease" OR pneumonia OR "shipping fever" OR morbidity OR mortality OR illness OR death OR pulls OR treatment)	1783<br>(6,107,832)
Q2	Beef AND	46,213
	(cow-calf OR pairs OR cows OR calves OR "beef cattle" OR beef OR cattle OR cow OR cows OR steer OR steers OR heifer OR calf OR calves OR stocker* OR feeder*) AND	27,754<br>(526,697)

	(colostrum OR colostral OR "colostrum management" OR "failure of passive transfer immunity" OR FPTI OR "failure of passive transfer" OR FPT OR survey OR management) AND	4014<br>(5,769,503)
	(BRD OR "bovine respiratory disease" OR pneumonia OR "shipping fever" OR morbidity OR mortality OR illness OR death OR pulls OR treatment)	1335<br>(6,107,832)
Q3	Beef AND	46,213
	(cow-calf OR pairs OR cows OR calves OR “beef cattle” OR beef OR cattle OR cow OR cows OR steer OR steers OR heifer OR calf OR calves OR stocker* OR feeder*) AND	27,754<br>(526,697)
	(creep OR "creep feeding" OR "calf supplement" OR “calf supplementation")	361 (10,436)
	(BRD OR "bovine respiratory disease" OR pneumonia OR "shipping fever" OR morbidity OR mortality OR illness OR death OR pulls OR treatment)	34<br>(6,107,832)
Proquest		
Dissertations		
Q1	Beef AND	122,762
	(cow-calf OR pairs OR cows OR calves OR “beef cattle” OR beef OR cattle OR cow OR cows OR steer OR steers OR heifer OR calf OR calves OR stocker* OR feeder*) AND	1,889,746<br>11,738<br>(NOFT-anywhere but full text)
	(nutrition OR "body condition score" OR BCS OR "fetal programming" OR "energy supplementation" OR "protein supplementation" OR "maternal nutrition" OR "offspring development" OR "developmental programming" OR vitamin* OR mineral* OR water OR Vitaferst-Care OR Multimin OR survey OR management) AND	3,251,790<br>1592 (NOFT)
	(BRD OR "bovine respiratory disease" OR pneumonia OR "shipping fever" OR morbidity OR mortality OR illness OR death OR pulls OR treatment)	2,714,222<br>700 (NOFT)
Q2	Beef AND	122,762
	(cow-calf OR pairs OR cows OR calves OR “beef cattle” OR beef OR	1,889,746

August 2026 | PRISMA-ScR checklist

	cattle OR cow OR cows OR steer OR steers OR heifer OR calf OR calves OR stocker* OR feeder*) AND	11,738 (NOFT- anywhere but full text)
	(colostrum OR colostral OR "colostrum management" OR "failure of passive transfer immunity" OR FPTI OR "failure of passive transfer" OR FPT OR survey OR management) AND	698,228<br>966 (NOFT
	(BRD OR "bovine respiratory disease" OR pneumonia OR "shipping fever" OR morbidity OR mortality OR illness OR death OR pulls OR treatment)	2,714,222<br>366 (NOFT)
Q3	Beef AND	122,762
	(cow-calf OR pairs OR cows OR calves OR “beef cattle” OR beef OR cattle OR cow OR cows OR steer OR steers OR heifer OR calf OR calves OR stocker* OR feeder*) AND	1,889,746<br>11,738 (NOFT- anywhere but full text)
	(creep OR "creep feeding" OR "calf supplement" OR “calf supplementation")	5,737<br>29 (NOFT)
	(BRD OR "bovine respiratory disease" OR pneumonia OR "shipping fever" OR morbidity OR mortality OR illness OR death OR pulls OR treatment)	2,714,222<br>22 (NOFT)

Supplementary File S1b. Level 1 Relevance Screening Form

Objective: To exclude clearly irrelevant records based on titles and abstracts while retaining uncertain records for Level 2 relevance confirmation.

Question	Options	Comments
1. Does the record describe primary research or an eligible research synthesis conducted in North America, involving beef cattle, published in English or French, and published in 2000 or later?	☐ Yes<br>☐ No<br>☐ Not sure	General eligibility
2. Does the research apply or evaluate a nutritional intervention or management exposure, including colostrum management, in beef cows, breeding females, or pre-weaning calves?	☐ Yes<br>☐ No<br>☐ Not sure	Intervention or exposure
3. Does the record report, or appear likely to report, at least one eligible outcome?	☐ Yes<br>☐ No<br>☐ Not sure	Eligible outcomes include BRD-specific morbidity or mortality; pneumonia or respiratory disease; BRD-specific treatment; general morbidity; first pull; general antimicrobial treatment; crude mortality; eligible secondary outcomes; or eligible tertiary outcomes.

Promotion logic
Promote the record to Level 2 if all three questions are answered Yes or Not sure. Exclude at Level 1 only when at least one question is answered No.

Execution
Two reviewers independently screened a calibration sample of 50 records and resolved disagreements through discussion. After calibration, the remaining records were screened according to the review protocol.

<u>Definitions and decision rules</u>
Beef cows: Includes primiparous and multiparous breeding females.
Pre-weaning calves: Calves that have not been permanently separated from the dam.
Nutritional intervention or management exposure: Includes nutritional content and delivery logistics, such as colostrum-delivery methods, nutritional injections, creep feeding, maternal supplementation, and pasture-management comparisons with clearly differing nutritional consequences.
Neonatal period: Birth through seven days of age, inclusive. Do not exclude at Level 1 solely because the title or abstract does not clearly distinguish neonatal-only outcomes from later outcomes.

Outcome distinctions: First pull, repull, retreatment, general antimicrobial treatment, and BRD-specific treatment are distinct outcomes. Repull and retreatment alone are not eligible secondary outcomes. First pull is not combined with repull or retreatment when reported separately.
Outcome hierarchy: BRD-specific outcomes identify BRD, pneumonia, respiratory disease, respiratory mortality, or respiratory treatment based on a defined case criterion. Secondary outcomes include nonspecific morbidity, first pull, general antimicrobial treatment, crude mortality, and other eligible clinical outcomes. Tertiary outcomes include immunological markers, vaccine responses, and other mechanistic outcomes relevant to BRD. Reports containing tertiary outcomes only are retained for evidence mapping and assessed at Level 2.

Supplementary File S1c. Level 2 Relevance Confirmation Form

Purpose: To confirm full-text eligibility, identify linked reports, assign the report to the appropriate extraction pathway, and record the final outcome category.

**Question**	**Options**	**Comments**
1. Does the report meet the general eligibility criteria?	☐ Yes<br>☐ No<br>☐ Unclear	English or French; 2000 or later; North America; eligible beef-cattle population; eligible design.
2. Does the report evaluate an eligible nutritional intervention or management exposure, including colostrum management, in beef cows, breeding females, or pre-weaning calves?	☐ Yes<br>☐ No, no relevant intervention or exposure<br>☐ No, wrong population<br>☐ Unclear<br>☐ Other: __________	Confirm intervention and population relevance.
3. What is the study design? Check all that apply.	☐ Randomized controlled trial<br>☐ Nonrandomized controlled trial<br>☐ Challenge trial<br>☐ Controlled challenge trial<br>☐ Cross-sectional study<br>☐ Cohort study<br>☐ Case-control study<br>☐ Outbreak or case-series description<br>☐ Systematic or scoping review<br>☐ Other eligible design: __________<br>☐ Ineligible design	Record the most specific applicable design.
4. What is the nature of the intervention or exposure? Check all that apply.	☐ Specific diet provided to dam<br>☐ Nutritional supplement or management applied to dam<br>☐ Nutritional injection administered to dam<br>☐ Combination applied to dam<br>☐ Nutritional supplement or management applied to calf<br>☐ Nutritional injection administered to calf<br>☐ Combination applied to calf<br>☐ Supplement applied to dam and calf<br>☐ Colostrum management<br>☐ Other: __________	Multiple selections are permitted.

	☐ None of the above	
5. At what production stage was the intervention or exposure applied? Check all that apply.	☐ Neonatal period, birth through seven days of age, inclusive<br>☐ Pre-weaning calf after seven days of age<br>☐ Cow or first-calving heifer with a suckling calf<br>☐ Dry cow or pregnant breeding female<br>☐ Multiple stages<br>☐ Timing not reported<br>☐ None of the above	Intervention timing is distinct from outcome timing.
6. When were the relevant outcomes assessed? Check all that apply.	☐ Neonatal period only, birth through seven days of age, inclusive<br>☐ After seven days but before weaning<br>☐ Entire pre-weaning period<br>☐ Post-weaning receiving or backgrounding period<br>☐ Feedlot or finishing period<br>☐ Multiple periods<br>☐ Timing not reported<br>☐ None of the above	Neonatal-only outcomes are excluded from the clinical synthesis.
7. Which BRD-specific outcomes are reported? Check all that apply.	☐ BRD morbidity<br>☐ BRD mortality<br>☐ Pneumonia or respiratory-disease morbidity<br>☐ Respiratory mortality<br>☐ BRD-specific treatment based on a defined case criterion<br>☐ No BRD-specific outcome	General sickness or antimicrobial use is not BRD-specific unless linked to a respiratory case definition.
8. Which secondary outcomes are reported? Check all that apply.	☐ General morbidity<br>☐ First pull or undifferentiated initial treatment<br>☐ General antimicrobial treatment<br>☐ Crude or all-cause mortality<br>☐ Other eligible secondary outcome: ___________<br>☐ No secondary outcome	First pull remains distinct from repull and retreatment.
9. Are repull or retreatment outcomes reported?	☐ Repull<br>☐ Retreatment	These are charted separately and are not combined with first pull.

	☐ Neither	
10. Which tertiary outcomes are reported? Check all that apply.	☐ Humoral response to BRD vaccination<br>☐ Cell-mediated response to BRD vaccination<br>☐ Response to BRD pathogen challenge<br>☐ Innate immune marker relevant to BRD<br>☐ Other mechanistic or tertiary outcome: __________<br>☐ No tertiary outcome	Tertiary-only reports are retained for evidence mapping.
11. Does this report describe the same underlying study as another retrieved report?	☐ No<br>☐ Yes; linked report identifier: __________<br>☐ Unclear	Link reports before assigning a unique study identifier.
12. What is the final full-text decision?	☐ Eligible for full clinical-outcome extraction<br>☐ Eligible for tertiary evidence mapping only<br>☐ Excluded<br>☐ Pending clarification	Tertiary-only reports are not excluded when otherwise eligible.
13. What is the final mutually exclusive outcome category?	☐ Study reporting BRD-specific outcomes<br>☐ Study reporting secondary outcomes only<br>☐ Study reporting tertiary outcomes only<br>☐ Excluded	A BRD-specific study may also report secondary or tertiary outcomes but remains in the BRD-specific category.
14. If excluded, record one principal exclusion reason.	☐ Wrong population<br>☐ Wrong intervention or exposure<br>☐ Wrong outcome<br>☐ Outcomes assessed exclusively during the neonatal period<br>☐ Wrong geographic setting<br>☐ Not published in English or French<br>☐ Published before 2000<br>☐ Ineligible study design<br>☐ Duplicate report or record<br>☐ Full text unavailable<br>☐ Other: __________	Use the finalized Figure 1 categories.

Promotion and classification logic

Full clinical-outcome extraction: Retain reports containing at least one BRD-specific or secondary outcome and meeting all other eligibility criteria.

Tertiary evidence mapping: Retain reports containing tertiary outcomes only, assign them to the tertiary-only category, and chart them separately. Do not include them in the synthesis of BRD-specific or secondary clinical outcomes.

Exclude reports with no eligible intervention or exposure, population, design, timing, or outcome, or reports failing another eligibility criterion.

Link multiple reports of the same underlying study before final categorization. In the completed review, no multiple reports of the same underlying study were identified; therefore, 45 included reports represented 45 unique studies.

Supplementary File S1d. Data Extraction Form

Complete one form for each unique included study. Link associated reports before extraction.

A. Study identification

Field	Extracted information
Study identifier	
Associated report citation(s)	
Country and region	
Publication year	
Language	English or French
Study design	
Funding source	
Conflict-of-interest statement	

B. Population and setting

Field	Extracted information
Population receiving intervention or exposure	
Population assessed for outcomes	
Eligibility criteria used by the study	
Sample size for intervention or exposure	
Sample size for outcome assessment	
Intervention setting	
Outcome-assessment setting	
Unit of allocation	
Unit of analysis	

C. Intervention or exposure

Field	Extracted information
Intervention or exposure name	
Nutrient or management component	
Dose, concentration, or formulation	
Comparator or reference category	
Timing of initiation	
Duration	
Co-interventions	
Compliance or exposure measurement	

D. Outcome timing and hierarchy

Field	Extracted information
Outcome-assessment period	
Neonatal period involved	Birth through seven days of age, inclusive
BRD-specific outcome present	☐ Yes ☐ No
Secondary outcome present	☐ Yes ☐ No
Tertiary outcome present	☐ Yes ☐ No
Final mutually exclusive category	☐ Study reporting BRD-specific outcomes<br>☐ Study reporting secondary outcomes only

	☐ Study reporting tertiary outcomes only

E. Clinical outcomes

Field	Extracted information
BRD morbidity	Definition, numerator, denominator, unit, follow-up period
BRD mortality	Definition, numerator, denominator, unit, follow-up period
Pneumonia or respiratory disease	Definition, numerator, denominator, unit, follow-up period
First pull	Extract separately
Repull	Extract separately; do not combine with first pull
Retreatment	Extract separately; do not combine with first pull
General antimicrobial treatment	Extract separately
BRD-specific treatment	Extract separately and record case criterion
General morbidity	
Crude or all-cause mortality	

F. Secondary and tertiary outcomes

Field	Extracted information
Performance outcome	
Serum immunoglobulin or passive-transfer outcome	Analyte, threshold, units, sampling age
Vaccine-response outcome	
Challenge-response outcome	
Innate or cell-mediated immune outcome	
Other mechanistic outcome	

G. Effect estimates and analysis

Field	Extracted information
Outcome	
Exposure or intervention category	
Reference category	
Effect measure	
Point estimate	
95% confidence interval	
P value	
Crude or adjusted	
Adjustment variables	
Statistical model	
Clustering or repeated measures	
Numerator and denominator	
Direction of comparison	
Notes on duplicate or alternative analyses	

H. Reporting notes

Field	Extracted information

Zero-event interpretation	If no events occurred in any group, record that no comparative effect was estimable.
Causal language	Prefer associated with, related to, reported a lower frequency, or reported a between-group difference.
Missing information	Record as not reported; do not infer.
Reviewer notes	

Review rules

Language eligibility: English or French.

The neonatal period is birth through seven days of age, inclusive.

First pull, repull, retreatment, general antimicrobial treatment, and BRD-specific treatment are extracted as distinct outcomes. First pull is not combined with repull or retreatment when reported separately.

Reports containing tertiary outcomes only are retained for evidence mapping and charted separately but are not included in the synthesis of BRD-specific or secondary clinical outcomes.

No multiple reports of the same underlying study were identified in the completed review; therefore, 45 included reports represented 45 unique studies.

Supplementary File S1e. PRISMA-ScR Checklist

A rapid scoping review of nutritional management of beef cows and pre-weaning calves and the occurrence of bovine respiratory disease in pre- and post-weaning calves in North America

Section	Item	PRISMA-ScR requirement	Reported on	Status	Assessment and remaining action
TITLE	1 Title	Identify the report as a scoping review.	Title	Complete	The title identifies the work as a rapid scoping review.
ABSTRACT	2 Structured summary	Provide a structured summary covering background, objectives, eligibility, evidence sources, charting, results, and conclusions.	Abstract; Simple Summary	Complete	The abstract reports the background, objective, eligibility criteria, six databases and grey-literature sources, charting and verification by one reviewer using a pre-tested form, narrative synthesis without formal critical appraisal, study counts, main findings, and conclusions.
INTRODUCTION	3 Rationale	Describe the rationale and why a scoping-review approach is appropriate.	Section 1 (Introduction)	Complete	The Introduction establishes the burden of BRD, the diversity and complexity of nutrition and colostrum evidence, heterogeneous designs and outcomes, evidence gaps, and the rationale for mapping the field.
INTRODUCTION	4 Objectives	State the review questions and objectives with population, concept, and context.	Section 1 (Introduction)	Complete	The objective and subquestions identify beef cows and pre-weaning calves, nutrition and colostrum management, BRD morbidity or mortality before and after weaning, and the North American context.
METHODS	5 Protocol and registration	Indicate whether a protocol exists and where it can be accessed.	Section 1 (Introduction); Section 2.1	Complete	The manuscript reports a protocol deposited in the University of Saskatchewan Digital Repository and provides a persistent repository link.
METHODS	6 Eligibility criteria	Specify source characteristics used as eligibility criteria and provide rationales.	Section 2.2 (2.2.1–2.2.4); Supplementary Files S1b and S1c	Complete	PICOS and CoCoPop criteria cover populations, interventions, comparators, outcomes, timing, designs, geography, language, publication period, and exclusions. The manuscript explains key restrictions, including publication date, language, geography, and neonatal-only outcomes.
METHODS	7 Information sources	Describe all information sources and the most recent search date.	Sections 2.3 and 3.1; Supplementary File S1a	Complete	Six bibliographic databases, dissertations, hand searches, proceedings, newsletters, Google, and citation searching are described. The initial electronic searches were conducted on 7 October 2025 and were updated on 30 August 2026; S1a contains source-specific details and retrieval counts for both searches.
METHODS	8 Search	Present the full electronic search strategy for at least one database, including limits.	Sections 2.3 and 3.1; Supplementary File S1a (detailed search strings and staged retrieval counts)	Complete	The Methods explain development and combination of search terms and direct readers to S1a. S1a presents the detailed database-specific search strings, fields, limits, retrieval counts, and search date.
METHODS	9 Selection of sources of evidence	State the process for screening and determining eligibility.	Sections 2.1, 2.4 and 2.5; Supplementary Files S1b and S1c	Complete	Two reviewers independently assessed a calibration sample of 50 citations and agreed on all 50 decisions, corresponding to 100% observed agreement. One reviewer then screened the remaining 5,435 titles and abstracts and assessed full texts, with ambiguous decisions resolved through discussion.
METHODS	10 Data charting process	Describe the charting method, calibration, reviewer roles, verification, and investigator contact.	Section 2.6; Supplementary File S1d	Complete	A pre-tested extraction form was used. One reviewer extracted all data and subsequently verified the completed dataset against source reports. Additionally, another reviewer independently verified a subset of data extraction from each included paper. No study investigators were contacted.
METHODS	11 Data items	List and define variables sought and assumptions or simplifications.	Sections 2.2 and 2.6; Supplementary Files S1b, S1c and S1d	Complete	The manuscript defines BRD-specific, secondary, tertiary, surrogate, and contextual outcomes and identifies study characteristics, intervention details, timing, setting, genetics, vaccination, baseline nutritional status, and intervention-complexity variables.
METHODS	12 Critical appraisal of individual sources (optional)	If appraisal was conducted, report rationale and methods.	Section 2.7	Not applicable, explicitly reported	The Methods state that no formal critical appraisal or study-level risk-of-bias assessment was undertaken.

Section	Item	PRISMA-ScR requirement	Reported on	Status	Assessment and remaining action
METHODS	13 Synthesis of results	Describe methods for handling and summarizing charted data.	Section 2.7	Complete	Data were compiled and narratively synthesized by intervention type, production stage, outcome type, and assessment timing. The reasons for not conducting meta-analysis are reported.
RESULTS	14 Selection of sources of evidence	Report numbers screened, assessed, included, and excluded, ideally with a flow diagram.	Section 3.1; Figure 1	Complete	The revised flow diagram reports 11,438 database records and 27 records from other sources; 5,980 duplicate records removed; 5,485 records screened; 5,294 records excluded at title-and-abstract screening; 191 reports sought and assessed; 0 reports not retrieved; 144 full-text reports excluded with reasons; and 47 studies included. All totals and exclusion subcategories reconcile.
RESULTS	15 Characteristics of sources of evidence	Present charted characteristics for each source and provide citations.	Sections 3.2–3.5; Tables 1–4; Supplementary Tables S2 and S3	Complete	Study designs, populations, interventions, settings, outcomes, sample sizes, treatment findings, and other design characteristics are reported in the main and supplementary tables with citations.
RESULTS	16 Critical appraisal within sources (optional)	If appraisal was conducted, present appraisal results.	Not applicable	Not applicable	No formal critical appraisal was conducted.
RESULTS	17 Results of individual sources of evidence	Present relevant charted data for each included source.	Sections 3.3–3.6; Tables 1–4; Supplementary Tables S2 and S4–S6	Complete	Study-level findings are reported for roughage, maternal macronutrients, creep feeding, trace minerals, colostrum management, IgG, and secondary or tertiary outcomes.
RESULTS	18 Synthesis of results	Summarize charting results in relation to the review questions.	Sections 3.1–3.6	Complete	Results are synthesized by intervention and outcome category and include cross-study summaries of setting, disease frequency, statistical reporting, randomization, blinding, repeated measures, and commingling.
DISCUSSION	19 Summary of evidence	Summarize main results, concepts, evidence types, and relevance to key groups.	Section 4 (Discussion), including Sections 4.1 and 4.2	Complete	The Discussion presents four principal findings, distinguishes direct from indirect evidence, interprets protein, trace minerals, creep feeding, colostrum and IgG, and links heterogeneity to intervention components, context, effect modifiers, and implementation settings.
DISCUSSION	20 Limitations	Discuss limitations of the scoping-review process.	Section 4 (Discussion), limitations paragraph	Complete	The review limitations include no formal risk-of-bias assessment, single-reviewer screening after a duplicate calibration sample, possible missed studies or screening errors, focused extraction and synthesis, and uncertainty about the direction and magnitude of resulting effects.
DISCUSSION	21 Conclusions	Provide an overall interpretation and implications or next steps.	Section 5 (Conclusions)	Complete	The Conclusions identify promising intervention areas, explain why specific recommendations remain premature, and call for adequately powered commercial trials with prespecified BRD outcomes, effect sizes, and confidence intervals.
FUNDING	22 Funding	Describe funding for the review and included evidence and the role of funders.	Funding statement (back matter)	Complete	The review funder and grant number are reported. The manuscript describes the funder-convened panel, states that funding sources for included studies were not charted, and specifies that the funder had no role in selection, extraction, interpretation, writing, or submission.

Checklist citation: Tricco, A.C.; et al. PRISMA Extension for Scoping Reviews (PRISMA-ScR): Checklist and Explanation. Ann. Intern. Med. 2018, 169, 467-473. doi:10.7326/M18-0850.
