# Supplementary material for: A Rapid Scoping Review of Nutritional Management of Beef Cows and Pre-Weaning Calves and the Occurrence of Bovine Respiratory Disease in Pre- and Post-Weaning Calves in North America
Source: Animals (Basel). 2026 Oct 4;16(19):3116. doi: 10.3390/ani16193116 (PMC13663391; doi:10.3390/ani16193116)
Supplement: Supplementary file 1 [file animals-16-03116-s001.zip › Supplementary_Table_S2.pdf]

Supplementary Table S2. Descriptive Summary of Twenty-five Studies Reporting Secondary or Tertiary Outcomes Only

Author, year [citation]	Location	Intervention or exposure	Population sampled for intervention (Outcome assessment)	Total number studied for intervention (Outcome assessment)	Replicates for intervention (Outcome assessment)	Outcomes assessed (Post-weaning study period)
Baumgaertner et al., 2024 [67]	US (North Dakota)	Basal diet targeting 0.28 kg/d gain or the basal diet plus an energy/protein supplement targeting 0.79 kg/d gain	Breeding heifers (Pre- and post-weaning heifer calves)	100 F0 heifers (45 Pre- and post-weaning heifer calves)	2 pens of 16 per treatment (tx) group (gp)	Colostrum IgG, vaccinal titres to IBR-BVD-PI-3 in F1 heifers; P4, insulin
Beck et al., 2005 [68]	US (Arkansas)	Mineral supplement with no Se, inorganic, or organic Se	Cows (Post-weaning calves)	6 gps 20 cows each (3 steer calves per gp)	2 replicates (reps) each of 3 tx gps (3 gps 6 steers each, commingled)	Lymphocyte proliferation, macrophage phagocytosis, IFN, glutathione peroxidase
Caramalac et al., 2017 [69]	US (Florida)	Cu, Zn, Mn supplement in hydroxy or sulfate-oxide forms, Negative control	Pre-weaning calves (Post-weaning calves)	44	4, 4, and 3 reps Year 1; 4, 4, 4 Year 2 for control, hydroxy and sulfate sups	Haptoglobin and ceruloplasmin; cortisol
Cruz et al., 2025 [51]	US (Montana)	Sulfate, organic complexed or hydroxy-based Cu, Mn, Zn supplement	Cows (Post-weaning calves)	72 cows	Treatment delivered individually, (1 rep commingled)	Mortality (Calculated for period from birth to end of backgrounding)
Dewell et al., 2006 [10][1]	US (Nebraska)	Neonatal serum IgG concentration categorized	Neonatal calves (Pre- and post-weaning calves)	1,556 calves	1 Herd (N/R)	Morbidity and mortality (To slaughter)
Fulton et al., 2023 [70]	US (South Dakota)	Inorganic (sulfate) or organic (proteinate) sources of Cu and Zn sups	Cows (Pre-weaning calf)	287 cows, 245 calves	1 rep per tx (N/A)	Serum total protein at 24–72 h; Antibodies to IBR, BVD, BRSV (N/A)
Gamsjäger et al., 2023 [9][1]	Canada (Alberta)	Neonatal serum IgG concentration categorized	Neonatal calves (Pre-weaning calves)	420 calves	6 herds (N/A)	Morbidity, Mortality (To weaning)
Gaspers, 2018 [52]	US (North Dakota)	Basal TMR ad lib +/- additional corn at 2% BW	Cows (Neonatal calves)	47 cows	2 reps per tx (N/A)	Colostrum and serum IgG concentration (N/A)
Goulais et al., 2026 [65]	US (Arkansas)	Injectable trace mineral (Cu, Mn, Se, Zn) administered 28 d before	Pre-weaning calves (Post-weaning calves)	115 calves (59 heifers, 56 steers)	12 pens of 10 calves post-weaning	Crude morbidity and mortality (Post-weaning receiving period = 42 d)

Author, year [citation]	Location	Intervention or exposure	Population sampled for intervention (Outcome assessment)	Total number studied for intervention (Outcome assessment)	Replicates for intervention (Outcome assessment)	Outcomes assessed (Post-weaning study period)
		weaning or at weaning; negative control (no ITM)				
Gunter et al., 2003 [53]	US (Arkansas)	Inorganic or organic (seleno-yeast) Se supplement	Cows (Pre-weaning calves)	120 cows Y1	2 reps /tx gp	Mortality (N/A)
Hall et al., 2022 [54]	US (Oregon)	Se boluses trimesters 1, 2, or 3	Cows (Post-weaning calves)	84 cows (46 calves)	Individual tx N/R	Morbidity (To slaughter)
Hurlbert et al., 2024 [55]	US (North Dakota)	Vitamin-mineral sup during gestation commercial product	Breeding heifers (Neonatal heifer calves)	72 heifers	Treatment delivered individually, (1 rep commingled)	Neonatal serum IgG concentrations (N/A)
Moriel and Arthington, 2013 [71]	US (Florida)	Limit-fed creep	Pre-weaning calves (Post-weaning calves)	278 cow-calf pairs	2 (neg control), 3, and 3 reps	Serum anti-PRBC immunoglobulin titers and plasma concentrations of haptoglobin and ceruloplasmin
Muehlenbein et al., 2001 [13]	US (Nebraska)	Inorganic Cu (sulfate), organic Cu , or organic Cu and Zn sups	Breeding heifers (Pre-weaning calves)	Y1 = 75 heifers Y2 = 120	Tx delivered individually	Morbidity (N/A)
Murray et al., 2016 [56] 1	Canada (Alberta)	Colostrum administered to calves born from dystocia	Neonatal calves (Pre-weaning calves)	267 herds	N/A	Mortality (To weaning)
Petit and Berthiaume, 2006 [57]	Canada (Québec)	Ca salts of palm oil or flaxseed sup, or negative control	Cows (Pre-weaning calves)	Y1 = 119 cows Y2 = 105	3–4 reps per tx gp Y1 and 2	Mortality (N/A)
Ricks, 2018 [58]	US (South Carolina)	Rumen-protected unsaturated fatty acid sup	Cows (Neonatal calves)	56 cows	Tx delivered individually	Colostrum and serum IgG concentration (N/A)
Stokes et al., 2018 [59]	US (Illinois)	Repeated Cu, Se, Mn, and Zn injections during gestation at 205, 114, and 44 ± 26 days prepartum	Breeding heifers (Pre-weaning calves)	190 heifers	N/R	Morbidity (N/A)
Stalker et al., 2006 [60]	US (Nebraska)	1.Irrigated pasture vs hay<br>2. Protein sup (0.45 kg/day) in 2 X 2 factorial design	Cows (Neonatal calves)	Y1 = 136 cows, Y2 = 113, Y3 = 113	2 reps per tx gp	Neonatal serum IgG
Waldner and Uehlinger, 2017 [61] 1	Canada (Alberta, Saskatchewan)	Serum Vitamin A/E levels categorized	Neonatal calves (Pre-weaning calves)	150 herds, 899 calves	N/A	BRD morbidity, Morbidity, Mortality

Author, year [citation]	Location	Intervention or exposure	Population sampled for intervention (Outcome assessment)	Total number studied for intervention (Outcome assessment)	Replicates for intervention (Outcome assessment)	Outcomes assessed (Post-weaning study period)
Waldner and Rosengren, 2009 [11][1]	Canada (Alberta, Saskatchewan)	Neonatal serum IgG concentration categorized; Vitamin E/Selenium injections	Neonatal calves (Pre-weaning calves)	152 herds, 935 calves, 601 sampled @ 2–8 d old	N/A	BRD morbidity, Morbidity, Mortality
Wilcox et al., 2026 [66]	Canada (Saskatchewan)	Injectable trace mineral (Cu, Mn, Se, Zn) plus granular trace mineral, 4 wk pre-calving and 4 wk pre-breeding; control (granular trace mineral alone)	Cows (Pre-weaning calves)	ITM = 97, 97, 100 cows; control = 98, 100, 101 cows (years 1-3)	NR	Crude pre-weaning mortality (Pre-weaning period)
Wilson et al., 2016 [62]	US (Illinois)	Ground hay plus dried distillers' grains with solubles (DDGS) vs corn bran plus ground cornstalks fed to 100% and 125% of TDN requirements, respectively.	Cows (Pre- and post-weaning calves)	106 cows	5 reps per tx gp, (2 reps commingled)	Morbidity (To slaughter)
Wilson et al., 2015a [63]	US (Illinois)	Limit-fed ground mixed, cool-season grass hay, limit-fed corn coproducts and ground cornstalks differing in RUP and fat content.	Cows (Pre- and post-weaning calves)	191 cows	8 reps per tx gp, (2 reps commingled)	Morbidity, Mortality (To slaughter)
Wilson et al., 2015b [64]	US (Illinois)	DDGS sup (2.1 kg/cow/day) (CP = 23%; fat = 7%); Negative control.	Cows (Post-weaning calves)	251 cows	6 reps per tx, (3 reps commingled)	Morbidity (To slaughter)

Abbreviations: BoCoV = Bovine coronavirus; BoHV-1 = Bovine Herpesvirus-1; BPIV-3 = Bovine parainfluenzavirus-3; BRSV =Bovine Respiratory Syncytial Virus; BVDV = Bovine Viral Diarrhea Virus; Cu = Copper; IFN = interferon; Ig = Immunoglobulin; F0 = Foundational generation of breeding program; F1 = First generation crossbred; Gp = Group; Mn = Manganese; N/A = Not Applicable; N/R = Not Reported; PRBC = Porcine Red Blood Cell; Rep = Replicate; Se = Selenium; Sup = Supplement; Tx = Treatment; Zn = Zinc.

[1] Study design: Observational study. All citations not superscripted are controlled trials.

References

Baumgaertner, F.; Menezes, A.C.B.; Diniz, W.J.S.; Hurlbert, J.L.; Bochantin-Winders, K.A.; Underdahl, S.R.; Kirsch, J.D.; Dorsam, S.T.; McCarthy, K.L.; Ramirez-Zamudio, G.D.; et al. Effects of rate of body weight gain during the first trimester of gestation on beef heifer and offspring performance, concentrations of hormones and metabolites, and response to vaccination. *J. Anim. Sci.* **2024**, *102*, https://doi.org/10.1093/jas/skae193.

Beck, P.A.; Wistube, T.J.; Davis, M.E.; Gunter, S.A. Case study: Effects of feeding Supplemental Organic or Inorganic Selenium to Cow-Calf Pairs on Selenium Status and Immune Responses of Weaned Beef Calves. Prof. *Animal* Scientist 2005, 21, 114–120.

Caramalac, L.S.; Netto, A.S.; Martins, P.; Moriel, P.; Ranches, J.; Fernandes, H.J.; Arthington, J.D. Effects of hydroxychloride sources of copper, zinc, and manganese on measures of supplement intake, mineral status, and pre- and postweaning performance of beef calves. *J. Anim. Sci.* **2017**, *95*, 1739–1750, https://doi.org/10.2527/jas.2016.0934.

Cruz, V.A.; Marques, R.S.; Kvamme, K.; Limede, A.C.; Cidrini, F.A.A.; Cidrini, I.A.; Dos Santos Nascimento, K.; Mackey, S.J.; Cooke, R.F.; Farmer, C.; et al. Effects of maternal Cu, Mn, and Zn supplementation from different sources on physiological and productive responses of cows and their offspring. *J. Anim. Sci.* **2025**, *103*, https://doi.org/10.1093/jas/skae391

Dewell, R.D.; Hungerford, L.L.; Keen, J.E.; Laegreid, W.W.; Griffin, D.D.; Rupp, G.P.; Grotelueschen, D.M. Association of neonatal serum immunoglobulin G1 concentration with health and performance in beef calves. *J. Am. Vet. Med. Assoc.* **2006**, *228*, 914–921, https://doi.org/10.2460/javma.228.6.914.

Fulton, J.O.; Blair, A.D.; Underwood, K.R.; Daly, R.F.; Gonda, M.G.; Perry, G.A.; Wright, C.L. The effect of copper and Zinc Sources on Liver Copper and Zinc Concentrations and Performance of Beef Cows and Suckling Calves. *Vet. Sci.* **2023**, *10*, https://doi.org/10.3390/vetsci10080511.

Gamsjäger, L.; Haines, D.M.; Lévy, M.; Pajor, E.A.; Campbell, J.R.; Windeyer, M.C. Total and pathogen-specific serum Immunoglobulin G concentrations in neonatal beef calves, Part 2: Associations with health and growth. *Prev. Vet. Med.* **2023**, *220*, 105993, https://doi.org/10.1016/j.prevetmed.2023.105993.

Gaspers, J. The effects of maternal diet on passive transfer of immunity to the neonatal beef calf, and calf immune response to vaccination and immunostimulants. PhD dissertation. North Dakota State University, Fargo, North Dakota, US, 2018.

Goulais, M.E.; Snider, M.A.; Phillips, C.; Justice, S.M.; Powell, J.G.; Shelton, C.T.; Gourley, G.; Jones, R.C.; Rivera, J.D. Effects of Timing of Injectable Trace Mineral Administration on Beef Calf Performance and Health Following Simulated Marketing. Animals 2026, 16, 1430, https://doi.org/10.3390/ani16101430.Gunter, S.A.; Beck, P.A.; Phillips, J.K. Effects of supplementary selenium source on the performance and blood measurements in beef cows and their calves. *J. Anim. Sci.* **2003**, *81*, 856–864, https://doi.org/10.2527/2003.814856x.

Hall, J.A.; Isaiah, A.; McNett, E.R.L.; Klopfenstein, J.J.; Davis, T.Z.; Suchodolski, J.S.; Bobe, G. Supranutritional Selenium-Yeast Supplementation of Beef Cows during the Last Trimester of Pregnancy Results in Higher Whole-Blood Selenium Concentrations in Their Calves at Weaning, but Not Enough to Improve Nasal Microbial Diversity. *Animals* (Basel) 2022, 12, https://doi.org/10.3390/ani12111360.

Hurlbert, J.L.; Baumgaertner, F.; Bochantin-Winders, K.A.; Jurgens, I.M.; Sedivec, K.K.; Dahlen, C.R. Effects of Vitamin and Mineral Supplementation During Gestation in Beef Heifers on Immunoglobulin Concentrations in Colostrum and Immune Responses in Naturally and Artificially Reared Calves. *Vet. Sci.* **2024**, *11*, https://doi.org/10.3390/vetsci11120635.

Moriel, P.; Arthington, J.D. Effects of trace mineral-fortified, limit-fed preweaning supplements on performance of pre- and postweaned beef calves. *J. Anim. Sci.* **2013**, *91*, 1371–1380, https://doi.org/10.2527/jas.2012-5469.

Muehlenbein, E.L.; Brink, D.R.; Deutscher, G.H.; Carlson, M.P.; Johnson, A.B. Effects of inorganic and organic copper supplemented to first-calf cows on cow reproduction and calf health and performance. *J. Anim. Sci.* **2001**, *79*, 1650–1659, https://doi.org/10.2527/2001.7971650x.

Murray, C.F.; Fick, L.J.; Pajor, E.A.; Barkema, H.W.; Jelinski, M.D.; Windeyer, M.C. Calf management practices and associations with herd-level morbidity and mortality on beef cow-calf operations. *Animal* **2016**, *10*, 468–477, https://doi.org/10.1017/s1751731115002062.

Petit, H.V.; Berthiaume, R. Effect of feeding different sources of fat during gestation and lactation on reproduction of beef cows and calf performance. *Can. J. Anim. Sci.* **2006**, *86*, 235–243.

Ricks, R.E. The effects of late gestation supplementation of rumen protected fat on cow and calf serum fatty acids, passive immunity transfer and growth in beef calves. MSc Thesis. Clemson University, 2018.

Stalker, L.A.; Adams, D.C.; Klopfenstein, T.J.; Feuz, D.M.; Funston, R.N. Effects of pre- and postpartum nutrition on reproduction in spring calving cows and calf feedlot performance. *J. Anim. Sci.* **2006**, *84*, 2582–2589, https://doi.org/10.2527/jas.2005-640.

Stokes, R.S.; Ireland, F.A.; Shike, D.W. Influence of repeated trace mineral injections during gestation on beef heifer and subsequent calf performance. *Transl. Anim. Sci.* **2018**, *3*, 493–503, https://doi.org/10.1093/tas/txy105.

Waldner, C.L.; Rosengren, L.B. Factors associated with serum immunoglobulin levels in beef calves from Alberta and Saskatchewan and association between passive transfer and health outcomes. *Can. Vet. J.* **2009**, *50*, 275–281.

Waldner, C.L.; Uehlinger, F. Factors associated with serum vitamin A and vitamin E concentrations in beef calves from Alberta and Saskatchewan and the relationship between vitamin concentrations and calf health outcomes. *Can. J. Anim. Sci.* **2017**, *97*, 65–82.

Wilcox, C.; Damiran, D.; Larson, K.; Erickson, N.; Christensen, D.; Waldner, C.; Lardner, H.A. Effect of injectable trace mineral supplementation on grazing beef cow and calf performance in western Canada. *Can. J. Anim. Sci.* **2026**, *106*, 1–9, https://doi.org/10.1139/cjas-2025-0088.

Wilson, T.B.; Faulkner, D.B.; Shike, D.W. Influence of late gestation drylot rations differing in protein degradability and fat content on beef cow and subsequent calf performance. *J. Anim. Sci.* **2015**, *93*, 5819–5828, https://doi.org/10.2527/jas.2014-8805.

Wilson, T.B.; Faulkner, D.B.; Shike, D.W. Influence of prepartum dietary energy on beef cow performance and calf growth and carcass characteristics. *Livest. Sci.* **2016**, *184*, 21–27, https://doi.org/10.1016/j.livsci.2015.12.004

Wilson, T.B.; Schroeder, A.R.; Ireland, F.A.; Faulkner, D.B.; Shike, D.W. Effects of late gestation distillers grains supplementation on fall-calving beef cow performance and steer calf growth and carcass characteristics. *J. Anim. Sci.* **2015**, *93*, 4843–4851, https://doi.org/10.2527/jas.2015-9228.
