# Supplementary material for: A Rapid Scoping Review of Nutritional Management of Beef Cows and Pre-Weaning Calves and the Occurrence of Bovine Respiratory Disease in Pre- and Post-Weaning Calves in North America
Source: Animals (Basel). 2026 Oct 4;16(19):3116. doi: 10.3390/ani16193116 (PMC13663391; doi:10.3390/ani16193116)
Supplement: Supplementary file 1 [file animals-16-03116-s001.zip › Supplementary_Table_S4.pdf]

Supplementary Table S4. Macronutrient Interventions Reporting Secondary Outcomes

Author, year [citation]	Intervention	Intervention Timing (Duration)	Setting of intervention (Setting of outcome assessment)	Population-Outcome (Alternate definition for morbidity if reported)	Comparator (Incidence of outcome in control group)	Results (*P* value)
**ROUGHAGE-BASAL DIETS TO COWS**						
Wilson et al., 2015a [63]	Mixed cool season grass hay vs corn coproducts and ground cornstalks.	88 ± 11 d prepartum (88 d)	Res c-c farm (Res c-c farm)	Pre-weaning morbidity	Across tx gps Hay-based ration (13.10%)	*P* = 0.90
			Res c-c farm (Res c-c farm)	Pre-weaning mortality	Across tx gps Hay-based ration (4.50%)	*P* = 0.80
			Res c-c farm (Res feedlot)	Post-weaning morbidity	Across tx gps Hay-based ration (25.9%)	*P* = 0.85
			Res c-c farm (Res feedlot)	Post-weaning mortality	Across tx gps Hay-based ration (2.8%)	*P* = 0.68
Wilson et al., 2016 [62]	Ground hay plus Dried Distillers' Grains with Solubles (DDGS) vs corn bran plus ground cornstalks fed to 100% TDN vs 125% respectively.	83 ± 10 d prepartum (83 d)	Res c-c farm (Res c-c farm)	Pre-weaning morbidity	Across tx gps 100% TDN ration (0)	*P* = 0.27
			Res c-c farm (Res feedlot)	Post-weaning morbidity	Across tx gps 100% TDN ration (36.0%)	*P* = 0.93
**MACRONUTRIENTS TO COWS**						
Wilson et al., 2015b [64]	2.1 kg/cow/day DM Dried Distillers' Grains with Solubles (CP = 23%; fat = 7%); Negative control.	Mid-June - mid-August in fall calving herd (2 mo)	Res c-c farm (Res feedlot)	Post-weaning morbidity	Negative control (38%)	*P* = 0.62
Petit and Berthiaume, 2006 [57]	Calcium salts of palm oil (Megalac™); Flaxseed; Negative control	1 mo prepartum, to turnout	Res c-c farm (Res c-c farm)	Pre-weaning mortality	Across tx gps Negative control (19.7%)	*P* = 0.02 Increased in Controls relative to tx gps

Some studies employed a factorial study design and therefore the same investigation may be cited presenting different contrasts across nutrient topic sub-headings.

Abbreviations:

Across tx gp = significance of difference in the outcome is assessed across multiple treatment or control groups as opposed to contrasts; Com cow-calf = Commercial cow-calf farm; Com feedlot = Commercial feedlot; DDGS = dried distillers' grains with solubles; DM = Dry Matter; Gp = Group; Negative control = No intervention/treatment applied; Res cow-calf = Research cow-calf farm, i.e. operated by university or research facility; Res feedlot = Research feedlot; TDN = Total Digestible Nutrients; Tx = Treatment.

References

Petit, H.V.; Berthiaume, R. Effect of feeding different sources of fat during gestation and lactation on reproduction of beef cows and calf performance. *Can. J. Anim. Sci.* **2006**, *86*, 235–243.

Wilson, T.B.; Faulkner, D.B.; Shike, D.W. Influence of late gestation drylot rations differing in protein degradability and fat content on beef cow and subsequent calf performance. *J. Anim. Sci.* **2015**, *93*, 5819–5828, https://doi.org/10.2527/jas.2014-8805.

Wilson, T.B.; Faulkner, D.B.; Shike, D.W. Influence of prepartum dietary energy on beef cow performance and calf growth and carcass characteristics. *Livest. Sci.* **2016**, *184*, 21–27, https://doi.org/10.1016/j.livsci.2015.12.004

Wilson, T.B.; Schroeder, A.R.; Ireland, F.A.; Faulkner, D.B.; Shike, D.W. Effects of late gestation distillers grains supplementation on fall-calving beef cow performance and steer calf growth and carcass characteristics. *J. Anim. Sci.* **2015**, *93*, 4843–4851, https://doi.org/10.2527/jas.2015-9228.
