# Supplementary material for: A Rapid Scoping Review of Nutritional Management of Beef Cows and Pre-Weaning Calves and the Occurrence of Bovine Respiratory Disease in Pre- and Post-Weaning Calves in North America
Source: Animals (Basel). 2026 Oct 4;16(19):3116. doi: 10.3390/ani16193116 (PMC13663391; doi:10.3390/ani16193116)
Supplement: Supplementary file 1 [file animals-16-03116-s001.zip › Supplementary_Table_S5.pdf]

Supplementary Table S5. Trace-Mineral and Vitamin Interventions Reporting Secondary Outcomes

Author, year [citation]	Intervention	Timing of intervention initiation (Duration)	Setting of intervention application (Setting of outcome assessment)	Population-Outcome	Comparator (Incidence of outcome in control group)	(Treatment effect) *P* value	Comments/ Other health-related outcomes
**SELENIUM SUPPLEMENTATION TO COWS**							
Gunter et al., 2003 [53]	26 mg of supplemental Se/kg of free-choice mineral from sodium selenite, or 26 mg of supplemental Se/kg of a free-choice mineral from seleno-yeast, Negative control.	Dec. Year 1 (42 wk)	Res c-c farm (Res c-c farm)	Pre-weaning mortality	(N/R)	Across tx gps $P > 0.16$	Glutathione peroxidase
Hall et al., 2022 [54]	Once-weekly Se-yeast boluses containing 105 mg of Se, at start of trimesters 1, 2, or 3, Negative control.	First/second/third trimesters (3 mo)	Res c-c farm (Com feedlot)	Post-weaning morbidity	(N/R)	N/R "Treatment did not improve health in the feedlot"	
**COPPER AND OTHER TRACE MINERAL SUPPLEMENTATION TO COWS**							
Cruz et al., 2025 [51]	Cu, Mn, Zn in sulfate, organic complexed, or hydroxy form supplement (sup).	195 ± 5 d gestation, to calving (91 d)	Res c-c farm (Res feedlot)	Post-weaning mortality	Sulfate-based mineral (8.3%)	Across tx gps $P = 0.98$	calf IgG, cortisol, NEFA, β-HB, serum abs to IBR BVD PI3
Muehlenbein et al., 2001 [13]	Inorganic Cu sulfate; Organic Cu (AvailaCu 100, Zinpro); or Organic Cu plus Zn, AvailaCu 100 mg Cu plus AvailaZn 400 mg Zn, Zinpro) sups; Negative control	45 d prepartum to 60 d postpartum (110 d)	Res c-c farm (Res c-c farm)	Pre-weaning morbidity	Negative control (44.00%)	Across tx gps $P > 0.10$	
Stokes et al., 2018 [59]	Combined Cu, Se, Mn, Zn injection, Negative control	205, 114, 44 ± 26 d prepartum after 3 previous injections 90 d apart	Res c-c farm (Res c-c farm)	Pre-weaning morbidity	Negative control (9.40%)	$P = 0.43$	
			Res c-c farm (Res c-c farm)	Pre-weaning mortality	Negative control (10.5%)	$P = 0.80$	

Author, year [citation]	Intervention	Timing of intervention initiation (Duration)	Setting of intervention application (Setting of outcome assessment)	Population-Outcome	Comparator (Incidence of outcome in control group)	(Treatment effect) *P* value	Comments/ Other health-related outcomes
Wilcox et al., 2026 [66]	Injectable trace mineral (Cu, Mn, Se, Zn) plus granular 2:1 trace mineral, Control (granular 2:1 trace mineral alone)	4 wk pre-calving and 4 wk pre-breeding, each of 3 years	Res c-c farm (Res c-c farm)	Pre-weaning mortality	Control (N/R)	*Across tx gps* *P > 0.05*	Cow serum trace mineral concentrations, pregnancy rate, cow BW and BCS, calf birth and weaning weights also reported
**TRACE MINERALS AND VITAMIN SUPPLEMENTATION TO CALVES**							
Waldner and Uehlinger, 2017 [61]	Serum vitamin E (vit E) 1 µg/mL linear variable	1 mo old or less	Com c-c farm	Pre-weaning BRD morbidity		(RR = 0.87, 95% CI: 0.66–1.14) *P* = 0.31	MV GLMM with random intercept for herd
	Serum vit E categorized inadequate	1 mo old or less	Com c-c farm	Pre-weaning BRD morbidity	Serum vit E categorized as adequate	(RR = 0.57, 95% CI: 0.18–1.78) *P* = 0.33	
	Serum vit E 1 µg/mL linear variable	1 mo old or less	Com c-c farm	Pre-weaning mortality		(RR = 0.83, 95% CI: 0.53–1.52) *P* = 0.44	
	Serum vit E categorized inadequate	1 mo old or less	Com c-c farm	Pre-weaning mortality	Serum vit E categorized as adequate	(RR = 1.55, 95% CI: 0.49–4.95) *P* = 0.46	
	No injection vit E/Se at birth	1 mo old or less	Com c-c farm	Pre-weaning mortality	Injection vit E/Se at birth	(RR = 5.4, 95% CI: 1.85–15.8) *P* = 0.002	Increased risk of pre-weaning mortality in herds not injecting Vit E/Se, accounting for Additional variables[1]

Author, year [citation]	Intervention	Timing of intervention initiation (Duration)	Setting of intervention application (Setting of outcome assessment)	Population-Outcome	Comparator (Incidence of outcome in control group)	(Treatment effect) *P* value	Comments/ Other health-related outcomes
Waldner and Rosengren, 2009 [11]	No Injection at birth	1 mo old or less	Com c-c farm	Pre-weaning morbidity or mortality	Injection vit E/Se	Estimate based on dataset similar to Waldner and Uehlinger, 2017 [61]	GEE with binomial distribution and logit link
Waldner and Uehlinger, 2017 [61]	Serum vitamin A (vit A) 1 µg/mL linear variable	1 mo old or less	Com c-c farm	Pre-weaning BRD morbidity		(RR = 1.44; 95% CI: 0.81–2.57) *P* = 0.21	MV GLMM with random intercept for herd
	Serum vit A categorized inadequate	1 mo old or less		Pre-weaning BRD morbidity	Serum vit A categorized as adequate	(RR = 0.73; 95% CI: 0.36–1.50) *P* = 0.31	
	Serum vit A 1 µg/mL linear variable	1 mo old or less		Pre-weaning mortality		(RR = 0.43; 95% CI: 0.19–0.96) *P* = 0.04	
	Serum vit A categorized inadequate	1 mo old or less		Pre-weaning mortality	Serum vit A categorized as adequate	IRR = 1.31; 95% CI 0.57–3.04; P = 0.52	
Waldner et al., 2022 [19]	Vitamin E/selenium injection near birth	Near birth	Commercial cow-calf herds (commercial cow-calf herds)	Pre-weaning BRD treatment	No vitamin E/selenium injection near birth	Effect measure, estimate, 95% CI, and vitamin-specific *P* value not reported	Observational herd-level exposure; not retained in the published final multivariable BRD models. Unit of analysis: herd-level management exposure and herd-level proportion of calves treated for BRD.

Author, year [citation]	Intervention	Timing of intervention initiation (Duration)	Setting of intervention application (Setting of outcome assessment)	Population-Outcome	Comparator (Incidence of outcome in control group)	(Treatment effect) *P* value	Comments/ Other health-related outcomes
Waldner et al., 2022 [19]	Vitamin A/D injection near birth	Near birth	Commercial cow-calf herds (commercial cow-calf herds)	Pre-weaning BRD treatment	No vitamin A/D injection near birth	Effect measure, estimate, 95% CI, and vitamin-specific *P* value not reported	Observational herd-level exposure; not retained in the published final multivariable BRD models. Unit of analysis: herd-level management exposure and herd-level proportion of calves treated for BRD.
Goulais et al., 2026 [65]	Injectable trace mineral (Cu, Mn, Se, Zn) 28 d before weaning or at weaning (2 tx gps), Negative control (no ITM)	28 d before weaning or at weaning (single injection; 42 d post-weaning follow-up)	Res c-c farm (Res feedlot)	Post-weaning morbidity	Negative control (N/R)	*Across tx gps P = 0.34*	Mortality reported as minimal (n = 2); serum Cu, Mn, and Se increased with ITM; BRSV titres, haptoglobin, and hair cortisol also reported

Abbreviations: Across tx gp = Significance of difference in the outcome is assessed across multiple treatment or control groups as opposed to contrasts; β-HB = β-hydroxybutyrate; Com cow-calf = Commercial cow-calf farm; Com feedlot = Commercial feedlot; Cu = Copper; d = Day; Ig = Immunoglobulin; Mn = Manganese; Mo = Month; NEFA = non-esterified fatty acids; Res cow-calf = Research cow-calf farm, i.e. operated by university or research facility; Res feedlot = Research feedlot; Se = Selenium; Serum abs to IBR, BVD, PI3 = serum antibodies to Infectious Bovine Rhinotracheitis Virus, Bovine viral diarrhea virus types 1 and 2 and parainfluenza-3 virus; Vit E = Vitamin E; Vit A = Vitamin A; wk = Week ; Zn = Zinc.

Treatment effects: RR = Risk ratio; OR = Odds ratio; 95% CI = 95% Confidence intervals; MV GLMM = Multivariable generalized mixed models; GEE = generalized estimating equations. [1]Variables included in final multivariable model for calf death: serum vitamin A concentration quartile: lowest relative to other quartiles; calf age at sampling in d, serum IgG concentration, month of calving; cow age at calving; dam body condition score; assistance required at birth (Y/N); Se and Vit E administered at birth (Y/N).

References

Cruz, V.A.; Marques, R.S.; Kvamme, K.; Limede, A.C.; Cidrini, F.A.A.; Cidrini, I.A.; Dos Santos Nascimento, K.; Mackey, S.J.; Cooke, R.F.; Farmer, C.; et al. Effects of maternal Cu, Mn, and Zn supplementation from different sources on physiological and productive responses of cows and their offspring. *J. Anim. Sci.* **2025**, *103*, https://doi.org/10.1093/jas/skae391

Goulais, M.E.; Snider, M.A.; Phillips, C.; Justice, S.M.; Powell, J.G.; Shelton, C.T.; Gourley, G.; Jones, R.C.; Rivera, J.D. Effects of Timing of Injectable Trace Mineral Administration on Beef Calf Performance and Health Following Simulated Marketing. Animals 2026, 16, 1430, https://doi.org/10.3390/ani16101430.

Gunter, S.A.; Beck, P.A.; Phillips, J.K. Effects of supplementary selenium source on the performance and blood measurements in beef cows and their calves. *J. Anim. Sci.* **2003**, *81*, 856–864, https://doi.org/10.2527/2003.814856x.

Hall, J.A.; Isaiah, A.; McNett, E.R.L.; Klopfenstein, J.J.; Davis, T.Z.; Suchodolski, J.S.; Bobe, G. Supranutritional Selenium-Yeast Supplementation of Beef Cows during the Last Trimester of Pregnancy Results in Higher Whole-Blood Selenium Concentrations in Their Calves at Weaning, but Not Enough to Improve Nasal Microbial Diversity. *Animals* (Basel) 2022, 12, https://doi.org/10.3390/ani12111360.

Muehlenbein, E.L.; Brink, D.R.; Deutscher, G.H.; Carlson, M.P.; Johnson, A.B. Effects of inorganic and organic copper supplemented to first-calf cows on cow reproduction and calf health and performance. *J. Anim. Sci.* **2001**, *79*, 1650–1659, https://doi.org/10.2527/2001.7971650x.

Stokes, R.S.; Ireland, F.A.; Shike, D.W. Influence of repeated trace mineral injections during gestation on beef heifer and subsequent calf performance. *Transl. Anim. Sci.* **2018**, *3*, 493–503, https://doi.org/10.1093/tas/txy105.

Waldner, C.; Wilhelm, B.; Windeyer, M.C.; Parker, S.; Campbell, J. Improving beef calf health: frequency of disease syndromes, uptake of management practices following calving, and potential for antimicrobial use reduction in western Canadian herds. *Transl. Anim. Sci.* **2022**, *6*, txac151, https://doi.org/10.1093/tas/txac151.

Waldner, C.L.; Rosengren, L.B. Factors associated with serum immunoglobulin levels in beef calves from Alberta and Saskatchewan and association between passive transfer and health outcomes. *Can. Vet. J.* **2009**, *50*, 275–281.

Waldner, C.L.; Uehlinger, F. Factors associated with serum vitamin A and vitamin E concentrations in beef calves from Alberta and Saskatchewan and the relationship between vitamin concentrations and calf health outcomes. *Can. J. Anim. Sci.* **2017**, *97*, 65–82.

Wilcox, C.; Damiran, D.; Larson, K.; Erickson, N.; Christensen, D.; Waldner, C.; Lardner, H.A. Effect of injectable trace mineral supplementation on grazing beef cow and calf performance in western Canada. Can. J. Anim. Sci. 2026, 106, 1–9, https://doi.org/10.1139/cjas-2025-0088.
