# Supplementary material for: A Rapid Scoping Review of Nutritional Management of Beef Cows and Pre-Weaning Calves and the Occurrence of Bovine Respiratory Disease in Pre- and Post-Weaning Calves in North America
Source: Animals (Basel). 2026 Oct 4;16(19):3116. doi: 10.3390/ani16193116 (PMC13663391; doi:10.3390/ani16193116)
Supplement: Supplementary file 1 [file animals-16-03116-s001.zip › Supplementary_Table_S6.pdf]

Supplementary Table S6. Colostrum Management, Passive Transfer, and Immunoglobulin Outcomes

Study	Exposure or intervention	Timing/setting	Outcome and follow-up	Comparator/reference	Observed data	Effect estimate and analysis	Notes
**A. Clinical outcomes associated with colostrum-management practices**							
Murray et al., 2016 [56]	Routine colostrum administration to calves born after dystocia	At birth; commercial cow-calf operations	All-cause mortality from 7 days to weaning	No routine colostrum administration	Not reported	Adjusted absolute difference: 0.8 percentage points lower mortality; *P* = 0.02	Retained from the source extraction as a distinct practice from verification of suckling.
Gamsjäger et al., 2023 [9]	Colostrum intervention (any supplemental colostrum administered by bottle or tube)	First 24 h; 6 commercial cow-calf herds in western Canada	Pre-weaning treatment for any reason; pre-weaning all-cause mortality	No colostrum intervention	Treatment 9/26 (35%) vs 52/329 (16%); mortality 6/26 vs 7/363	Treatment: P = 0.02, univariable (multilevel model could not be fitted). Mortality: adjusted OR 6.1 (95% CI 1.5–24.5), P = 0.011 (Model 1); 7.4 (1.7–31.6), P = 0.007 (Model 2); 7.0 (2.5–40.2), P = 0.006 (Model 3); 12.7 (3.38–47.72), P < 0.0001 (Model 4); multilevel multivariable logistic regression with farm as a random effect	Colostrum intervention is given to calves judged to need assistance, so this association is open to confounding by indication; it is not evidence that intervention is harmful.
**B. Clinical outcomes associated with passive-transfer status**							
Gamsjäger et al., 2023 [9]	Failed transfer of passive immunity: serum IgG < 10 g/L	Sampled at 1–7 days; 6 commercial herds	Treatment for any reason before weaning	Serum IgG ≥ 10 g/L	FPT group: 20 calves; raw group-specific treatment counts not paired with the model estimate here	Unadjusted OR 7.9 (95% CI 2.7–23.7); *P* < 0.0001; univariable logistic regression	Comparator includes calves with inadequate transfer (10 to <24 g/L); it is not an "adequate" category.
Gamsjäger et al., 2023 [9]	Failed transfer of passive immunity: serum IgG < 10 g/L	Sampled at 1–7 days; 6 commercial herds	Pre-weaning all-cause mortality	Serum IgG ≥ 10 g/L	6/20 (30%) deaths in FPT group	Adjusted OR 18.5 (95% CI 3.7–93.4); P = 0.007; multivariable multilevel logistic regression	Do not pair the ≥24 g/L raw incidence with this model referent.

Study	Exposure or intervention	Timing/setting	Outcome and follow-up	Comparator/reference	Observed data	Effect estimate and analysis	Notes
Gamsjäger et al., 2023 [9]	Serum IgG < 24 g/L, combining failed and inadequate transfer categories	Sampled at 1–7 days; 6 commercial herds	Pre-weaning all-cause mortality	Adequate transfer: serum IgG ≥ 24 g/L	Adequate category: 4/317 (approximately 1%)	Adjusted OR 10.1 (95% CI 2.6–40.2); multivariable multilevel logistic regression	Distinct threshold analysis; not a duplicate of the < 10 g/L model.
Waldner and Rosengren, 2009 [11]	Each 10 g/L decline in serum IgG	Sampled at 2–8 days; commercial herds	Treatment for any therapeutic or prophylactic reason, >1 h after birth to earlier of 3 months or 30 June	Higher serum IgG (continuous model)	n = 601	Adjusted OR 1.23 (95% CI 1.03–1.47); *P* = 0.02; GEE logistic model	General treatment, not pneumonia-specific treatment.
Waldner and Rosengren, 2009 [11]	Each 10 g/L decline in serum IgG	Sampled at 2–8 days; commercial herds	All-cause mortality, >1 h after birth to earlier of 3 months or 30 June	Higher serum IgG (continuous model)	n = 601	Adjusted OR 1.60 (95% CI 1.10–2.30); *P* = 0.02; GEE logistic model	
Waldner and Rosengren, 2009 [11]	Serum IgG concentration	Sampled at 2–8 days; commercial herds	Treatment for pneumonia over the same follow-up	Higher serum IgG	27 calves treated for pneumonia in the larger cohort	No significant association; final exposure-specific estimate not reported	Kept separate from treatment for any reason.
Waldner and Rosengren, 2009 [11]	Categorical threshold analysis: serum IgG < 24 g/L	Sampled at 2–8 days; commercial herds	All-cause mortality	Serum IgG ≥ 24 g/L	n = 601	Adjusted OR 1.6 (95% CI 1.1–2.3); *P* = 0.02	Alternative parameterization of the continuous model, not an independent analysis.
Waldner and Rosengren, 2009 [11]	Categorical threshold analysis: serum IgG < 24 g/L	Sampled at 2–8 days; commercial herds	Treatment for any reason	Serum IgG ≥ 24 g/L	n = 601	Adjusted OR 1.5 (95% CI 1.0–2.3); *P* = 0.07	Alternative parameterization of the continuous model.
Dewell et al., 2006 [10]	FPT: serum IgG1 ≤ 800 mg/dL (≤ 8 g/L)	Sampled at 24–72 h; research cow-calf herd	At least one pre-weaning morbidity event	Adequate: serum IgG1 > 1,600 mg/dL (> 16 g/L)	FPT 48/221 (21.7%); adequate 121/1,201 (10.1%)	Adjusted OR 2.24 (95% CI 1.52–3.29); multivariable logistic regression	
Dewell et al., 2006 [10]	Marginal: serum IgG1 801–1,600 mg/dL	Sampled at 24–72 h; research cow-calf herd	At least one pre-weaning morbidity event	Adequate: serum IgG1 > 1,600 mg/dL	Marginal 18/134 (13.4%); adequate 121/1,201 (10.1%)	Adjusted OR 1.38 (95% CI 0.80–2.39); multivariable logistic regression	
Dewell et al., 2006	FPT: serum IgG1 ≤	Sampled at	Pre-weaning all-	Adequate: serum IgG1	FPT 19/221 (8.6%);	Adjusted OR 4.9	

Study	Exposure or intervention	Timing/setting	Outcome and follow-up	Comparator/reference	Observed data	Effect estimate and analysis	Notes
[10]	800 mg/dL	24–72 h; research cow-calf herd	cause mortality	> 1,600 mg/dL	adequate 20/1,203 (1.7%)	(95% CI 2.48–9.52); multivariable logistic regression	
Dewell et al., 2006 [10]	Marginal: serum IgG1 801–1,600 mg/dL	Sampled at 24–72 h; research cow-calf herd	Pre-weaning all-cause mortality	Adequate: serum IgG1 > 1,600 mg/dL	Marginal 3/135 (2.2%); adequate 20/1,203 (1.7%)	Adjusted OR 1.20 (95% CI 0.35–4.14); multivariable logistic regression	
Dewell et al., 2006 [10]	Neonatal serum IgG1 categories	Sampled at 24–72 h; feedlot follow-up	At least one feedlot morbidity event	Adequate: serum IgG1 > 1,600 mg/dL	Overall 175/1,513 (11.6%); FPT 20/202 (9.9%); marginal numerator not entered because the article narrative is internally inconsistent	No significant adjusted association	
Dewell et al., 2006 [10]	Neonatal serum IgG1 categories	Sampled at 24–72 h; feedlot follow-up	Feedlot mortality	Adequate: serum IgG1 >1,600 mg/dL	Overall 6/1,517 (0.4%); FPT 2/202 (1.0%); marginal 1/132 (0.8%); adequate 3/1,183 (0.25%)	Regression modelling not performed because only six deaths occurred	
Dewell et al., 2006 [10]	Alternative threshold: serum IgG1 ≤ 2,400 mg/dL	Sampled at 24–72 h	Pre-weaning morbidity	Serum IgG1 > 2,400 mg/dL	Not reported in this summary	Adjusted OR 1.6 (95% CI 1.19–2.28)	Alternative threshold analysis; separate from conventional categories.
Dewell et al., 2006 [10]	Alternative threshold: serum IgG1 < 2,400 mg/dL	Sampled at 24–72 h	Pre-weaning mortality	Serum IgG1 ≥ 2,400 mg/dL	Not reported in this summary	Adjusted OR 2.7 (95% CI 1.34–5.36)	Alternative threshold analysis.
**C. Immunoglobulin and passive-transfer outcomes from controlled trials**							
Brandão et al., 2020 [43]	Maternal calcium salts of soybean oil vs prilled fat	85 days prepartum	Colostrum IgG; calf serum IgG at 24 h	Prilled-fat control	Group means and units: see source report	Colostrum *P* = 0.02; serum *P* < 0.01	Continuous outcomes; *P* values are not effect estimates.
Cruz et al., 2025 [51]	Maternal Cu, Mn, and Zn supplied as sulfate, organic-complexed, or hydroxychloride sources	Late gestation to calving	Calf serum IgG at the source-reported sampling time	Across treatment groups	Group means and units: see source report	No treatment difference; *P* = 0.37	Do not describe as 24 h unless confirmed in source table.

Study	Exposure or intervention	Timing/setting	Outcome and follow-up	Comparator/reference	Observed data	Effect estimate and analysis	Notes
Gaspers, 2018 [52]	Basal ration vs additional corn	Day 110 of gestation to calving	Colostrum IgG; calf serum IgG at 1 day	Basal ration	Group means and units: see dissertation	*P* = 0.16; *P* = 0.86	
Hurlbert et al., 2024 [55]	Naturally derived maternal colostrum versus colostrum-replacement product administered to calves	At birth; serum at 24 h	Calf serum IgG, IgA, IgM, and total Ig at 24 h	Colostrum-replacement product	Maternal-colostrum group had higher concentrations	IgG *P* = 0.01; IgA and IgM *P* < 0.001; total Ig *P* = 0.01	Colostrum source comparison, not “suckling versus tube.”
Hurlbert et al., 2024 [55]	Gestational vitamin-mineral supplement vs control	Breeding through calving	Colostrum and calf serum IgG, IgA, and IgM at 24 h	No gestational supplement	Group means and units: see source report	No treatment effect; *P* ≥ 0.21	Separate factorial comparison from colostrum source.
Radunz et al., 2012 [39]	Hay, corn, or DDGS maternal diets	Late gestation	Neonatal serum IgG categories at 24–72 h	Across treatment groups	FPT < 8 g/L; marginal 8.01–15.99 g/L; adequate ≥ 16 g/L	*P* = 0.71, 0.86, and 0.82, respectively	
Ricks, 2018 [58]	Rumen-protected unsaturated fatty acids vs control	94 days prepartum	Colostrum IgG; calf serum IgG at 1 and 5 days	Negative control	Treatment group had higher IgG; means and units: see thesis	*P* = 0.007; *P* < 0.001; *P* = 0.02	
Stalker et al., 2006 [60]	Protein supplement and prebreeding pasture/hay factorial	Prepartum and prebreeding periods	Calf serum IgG at 24–72 h	No supplement; hay	Group means and units: see source report	*P* = 0.98 and *P* = 0.47	
Stephenson et al., 2023 [48]	Maternal Cu, Zn, and Mn sources	Late gestation to 11 days postpartum	Calf serum IgG, IgA, and IgM at 48 h	Across treatment groups	Group means and units: see source report	*P* = 0.57, 0.68, and 0.61	

Definitions: Gamsjäger et al.: failed transfer, serum IgG <10 g/L; inadequate transfer, serum IgG 10 to <24 g/L; adequate transfer, serum IgG ≥24 g/L. Waldner and Rosengren: thresholds assessed at ≤8, ≤16, and <24 g/L. Dewell et al.: FPT, serum IgG1 ≤800 mg/dL; marginal, 801–1,600 mg/dL; adequate, >1,600 mg/dL.

Abbreviations: BRD, bovine respiratory disease; CI, confidence interval; DDGS, dried distillers’ grains with solubles; FPT, failed transfer of passive immunity; GEE, generalized estimating equations; Ig, immunoglobulin; OR, odds ratio; P, probability value.

References

Brandão, A.P.; Cooke, R.F.; Schubach, K.M.; Rett, B.; Souza, O.A.; Schachtschneider, C.L.; Perry, G.A.; Arispe, S.A.; Jump, D.B.; Pohler, K.G.; et al. Supplementing Ca salts of soybean oil to late-gestating beef cows: impacts on performance and physiological responses of the offspring. *J. Anim. Sci.* **2020**, *98*, https://doi.org/10.1093/jas/skaa247.

Cruz, V.A.; Marques, R.S.; Kvamme, K.; Limede, A.C.; Cidrini, F.A.A.; Cidrini, I.A.; Dos Santos Nascimento, K.; Mackey, S.J.; Cooke, R.F.; Farmer, C.; et al. Effects of maternal Cu, Mn, and Zn supplementation from different sources on physiological and productive responses of cows and their offspring. *J. Anim. Sci.* **2025**, *103*, https://doi.org/10.1093/jas/skae391

Dewell, R.D.; Hungerford, L.L.; Keen, J.E.; Laegreid, W.W.; Griffin, D.D.; Rupp, G.P.; Grotelueschen, D.M. Association of neonatal serum immunoglobulin G1 concentration with health and performance in beef calves. *J. Am. Vet. Med. Assoc.* **2006**, *228*, 914–921, https://doi.org/10.2460/javma.228.6.914.

Gamsjäger, L.; Haines, D.M.; Lévy, M.; Pajor, E.A.; Campbell, J.R.; Windeyer, M.C. Total and pathogen-specific serum Immunoglobulin G concentrations in neonatal beef calves, Part 2: Associations with health and growth. *Prev. Vet. Med.* **2023**, *220*, 105993, https://doi.org/10.1016/j.prevetmed.2023.105993.

Gaspers, J. The effects of maternal diet on passive transfer of immunity to the neonatal beef calf, and calf immune response to vaccination and immunostimulants. PhD dissertation. North Dakota State University, Fargo, North Dakota, US, 2018.

Hurlbert, J.L.; Baumgaertner, F.; Bochantin-Winders, K.A.; Jurgens, I.M.; Sedivec, K.K.; Dahlen, C.R. Effects of Vitamin and Mineral Supplementation During Gestation in Beef Heifers on Immunoglobulin Concentrations in Colostrum and Immune Responses in Naturally and Artificially Reared Calves. *Vet. Sci.* **2024**, *11*, https://doi.org/10.3390/vetsci11120635.

Murray, C.F.; Fick, L.J.; Pajor, E.A.; Barkema, H.W.; Jelinski, M.D.; Windeyer, M.C. Calf management practices and associations with herd-level morbidity and mortality on beef cow-calf operations. *Animal* **2016**, *10*, 468–477, https://doi.org/10.1017/s1751731115002062.

Radunz, A.E.; Fluharty, F.L.; Relling, A.E.; Felix, T.L.; Shoup, L.M.; Zerby, H.N.; Loerch, S.C. Prepartum dietary energy source fed to beef cows: II. Effects on progeny postnatal growth, glucose tolerance, and carcass composition. *J. Anim. Sci.* **2012**, *90*, 4962–4974, https://doi.org/10.2527/jas.2012-5098.

Ricks, R.E. The effects of late gestation supplementation of rumen protected fat on cow and calf serum fatty acids, passive immunity transfer and growth in beef calves. MSc Thesis. Clemson University, 2018.

Stalker, L.A.; Adams, D.C.; Klopfenstein, T.J.; Feuz, D.M.; Funston, R.N. Effects of pre- and postpartum nutrition on reproduction in spring calving cows and calf feedlot performance. *J. Anim. Sci.* **2006**, *84*, 2582–2589, https://doi.org/10.2527/jas.2005-640.

Stephenson, E.L.; Rathert-Williams, A.R.; Kenny, A.L.; Nagy, D.W.; Shoemake, B.M.; McFadden, T.B.; Tucker, H.A.; Meyer, A.M. Effects of copper, zinc, and manganese source and inclusion during late gestation on beef cow-calf performance, mineral transfer, and metabolism. *Transl. Anim. Sci.* **2023**, *7*, txad097, https://doi.org/10.1093/tas/txad097.

Waldner, C.L.; Rosengren, L.B. Factors associated with serum immunoglobulin levels in beef calves from Alberta and Saskatchewan and association between passive transfer and health outcomes. *Can. Vet. J.* **2009**, *50*, 275–281.
